# Evaluation of proline-rich antimicrobial peptides as potential lead structures for novel antimycotics against *Cryptococcus neoformans*

**DOI:** 10.3389/fmicb.2023.1328890

**Published:** 2024-01-08

**Authors:** Alexandra Brakel, Thomas Grochow, Stefanie Fritsche, Daniel Knappe, Andor Krizsan, Simone A. Fietz, Gottfried Alber, Ralf Hoffmann, Uwe Müller

**Affiliations:** ^1^Institute of Bioanalytical Chemistry, Faculty of Chemistry and Mineralogy, Bioanalytical Chemistry, Leipzig University, Leipzig, Germany; ^2^Center for Biotechnology and Biomedicine, Leipzig University, Leipzig, Germany; ^3^Institute of Veterinary Anatomy, Histology and Embryology, Faculty of Veterinary Medicine, Leipzig University, Leipzig, Germany; ^4^Institute of Immunology/Molecular Pathogenesis, Faculty of Veterinary Medicine, Leipzig University, Leipzig, Germany

**Keywords:** antifungal peptide, fungal pathogen, intracellular mode-of-action, proline-rich antimicrobial peptide (PrAMP), scanning electron microscopy

## Abstract

**Background:**

Cryptococcosis and cryptococcal meningitis, caused by *Cryptococcus neoformans* infections, lead to approximately 180,000 deaths per year, primarily in developing countries. Individuals with compromised immune systems, e.g., due to HIV infection (AIDS) or chemotherapy, are particularly vulnerable. Conventional treatment options are often limited and can cause severe side effects. Therefore, this study aimed to investigate the antifungal effect of insect-derived proline-rich antimicrobial peptides (PrAMPs) against *C. neoformans*. These peptides are known for their low toxicity and their high efficacy in murine infection models, making them a promising alternative for treatment.

**Results:**

A preliminary screening of the minimal inhibitory concentrations (MICs) of 20 AMPs, including the well-known PrAMPs Onc112, Api137, and Chex1Arg20 as well as the cathelicidin CRAMP against the *C. neoformans* strains 1841, H99, and KN99α revealed promising results, with MICs as low as 1.6 μmol/L. Subsequent investigations of selected peptides, determining their influence on fungal colony-forming units, confirmed their strong activity. The antifungal activity was affected by factors such as peptide net charge and sequence, with stronger effects at higher net charges probably due to better intracellular uptake confirmed by confocal laser scanning microscopy. Inactive scrambled peptides suggest a specific intracellular target, although scanning electron microscopy showed that PrAMPs also damaged the cell exterior for a low proportion of the cells. Possible pore formation could facilitate entry into the cytosol.

## 1 Introduction

Fungal infections are widespread in populations of both developing and developed countries. Most people suffer from superficial fungal infections, usually of the skin, hair, or nails, during their lifetime that are not a major health concern (Bongomin et al., 2017; Fisher et al., 2020). Although the number of invasive fungal infections is significantly lower, it is of greater concern due to its high mortality rate, accounting for 1.5 million deaths per year. Species of *Candida*, *Pneumocystis*, *Aspergillus*, or *Cryptococcus* are responsible for approximately 90% of the deaths, primarily affecting individuals with compromised immune systems (Brown et al., 2012; Schmiedel and Zimmerli, 2016; Fisher et al., 2020). The prevalence of fungal infections, such as cryptococcosis or cryptococcal meningitis caused by *Cryptococcus neoformans*, has been steadily increasing since the 1950s, particularly in developing countries, due to HIV infection (AIDS) and modern immunosuppressive and invasive medical interventions, including chemotherapy (Hazen, 1995). Exposure to *C. neoformans* occurs primarily through inhalation of airborne organisms into the lungs, often transmitted by feces of various avian species (Cafarchia et al., 2006). The fungus primarily targets the central nervous system, causing inflammation of the brain and meninges (Park et al., 2009).

*C. neoformans* can be divided into three serotypes, i.e., serotype A (*C. n.* var. *grubii*), serotype D (*C. n. var. neoformans*), and a hybrid form (serotype AD). Differences in the capsular composition, melanin production, or maximum growth temperature, pose severe challenges in the development of antifungal drugs, as they must be effective against all strains and ideally should not induce resistance (Samarasinghe et al., 2018; Casadevall et al., 2019). It is also important that the antifungals are effective against all variants so that they can be used consistently regardless of the geographical occurrence of the serotypes, e.g., serotype A is predominant worldwide and serotype D is predominant in Europe (Martinez et al., 2001; Montoya et al., 2021).

The main drugs used for treatment are amphotericin B, fluconazole, and flucytosine in various combinations (Ngan et al., 2022). The administration of these antifungals is problematic due to their nephro- and neurotoxicity, as well as various gastrointestinal side effects (Muhaj et al., 2022). In addition, the rapid emergence of drug resistance is a growing challenge, underscoring the urgent need for the development of new classes of antifungal agents (Denning and Hope, 2010; Fisher et al., 2022).

In the quest for novel anti-infectives, antimicrobial peptides have attracted considerable attention in recent decades. Most of the antifungal peptides studied rely primarily on membrane disruption owing to their structural characteristics, such as positive charge and hydrophobicity (Sharma et al., 2017; Li et al., 2021; Sharma et al., 2022). However, a lytic mode of action always carries the risk of adverse effects, which is why intracellular mechanisms are preferred (Struyfs et al., 2021). Proline-rich AMPs (PrAMPs), which have been extensively studied *in vitro* and *in vivo* primarily for their activity against Gram-negative and Gram-positive bacteria over the past two decades, target the bacterial ribosome (Krizsan et al., 2014; Kolano et al., 2021). Short PrAMPs were originally isolated from insects, such as the milkweed bug (*Oncopeltus fasciatus*) or the honeybee (*Apis mellifera*), and further optimized for therapeutic applications by improving their activity against various bacteria and their protease (serum) stability (Knappe et al., 2010; Berthold et al., 2013; Knappe et al., 2016). Synthetic derivatives such as Api137, Onc112, or Chex1Arg20 bind to the chaperone DnaK and the 70S ribosome after active transport into the cell, thereby inhibiting protein folding, and translation during protein biosynthesis (Krizsan et al., 2014, 2015a,b; Brakel et al., 2022a,b). PrAMPs have proven to be valid lead compounds due to their high efficacy in murine infection models and their low adverse effects in animals, all of which can be attributed to their bacteria-specific uptake and targeting of the bacterial ribosome (Holfeld et al., 2015; Schmidt et al., 2017; Brakel et al., 2019). Little is known about the activity of PrAMPs against eukaryotic pathogens, e.g., fungi or protozoan parasites. In this context, we have devoted ourselves to the aforementioned fungus, *C. neoformans*, which is one of the main causes of death caused by invasive fungi. The mammalian PrAMPs SP-E (from porcine saliva) and Bac7 (from bovine neutrophils) are active against *C. neoformans* in the low micromolar range (Benincasa et al., 2004; Conti et al., 2013). Initial investigations have suggested the possibility of an intracellular mode of action against fungi, but to our knowledge, this has not been pursued further. Given the promising properties of insect-derived PrAMPs, this study aimed to investigate their activity against *C. neoformans* for the first time. While native apidaecin 1b, drosocin, and pyrrhocoricin were inactive against *C. neoformans*, a few optimized analogs of apidaecin and the designer peptide Chex1Arg20, also called ARV-1502, were highly active against three clinically relevant pathogenic *C. neoformans* strains with minimum inhibitory concentrations (MICs) of 4 mg/L (1.6 to 3 μmol/L). Fluorescence microscopy confirmed that these peptides enter the fungal cells, suggesting an intracellular mechanism. Interestingly, reverse sequences showed similar activities, challenging the ribosome as the primary target.

## 2 Materials and methods

### 2.1 Materials

Reagents were obtained from the following manufacturers: Carl Roth GmbH & Co. KG (Karlsruhe, Germany): Agar-Agar (Kobe I), ethanol (HPLC grade), d (+)-glucose monohydrate (> 99.5%), glutaraldehyde (25%, for electron microscopy) 1,1,1,3,3,3-hexamethyldisilazane (HMDS, ≥ 98%), paraformaldehyde, sodium dodecyl sulfate (SDS, >99.5%), and sodium hydroxide (> 98%); Degussa AG (Hanau, Germany): Osmium tetroxide (75%); Sigma Aldrich Chemie GmbH (Taufkirchen, Germany): Amphotericin B, 5(6)-carboxyfluorescein (for fluorescence), dimethyl sulfoxide (DMSO, ≥ 99.9%), disodium hydrogen phosphate (> 98%), fluconazole, flucytosine, glycerol-gelatin mounting medium (GG1), poly-L-lysine solution (0.01%), sodium dihydrogen phosphate (>99%), thiazolyl blue tetrazolium bromide (MTT; ≥ 97.5%), and Triton X-100; Thermo Fisher Scientific Inc. (Darmstadt, Germany): Gibco® DMEM/F-12 medium, Gibco® PBS, Gibco® Penicillin–Streptomycin (10,000 U/mL), and Gibco® Trypsin–EDTA (0.5%); VWR International GmbH (Darmstadt, Germany): peptone (from casein).

Porcine blood (fattening hybrid pig, German Landrace x Pietrain) for the determination of hemolytic activity was obtained from the Veterinary Faculty of the Leipzig University. The blood collection was performed in accordance with the German Animal Welfare legislation and approved by the Landesdirektion Sachsen (reference number 25–5131/556/16).

### 2.2 Peptide synthesis

Peptides were synthesized in-house using Fmoc/^t^Bu-chemistry on Rink amide or Wang resin and purified by RP-HPLC using a linear acetonitrile gradient in the presence of 0.1% TFA as previously reported (Knappe et al., 2010; Berthold et al., 2013). Masses were confirmed by ESI-MS and the purities (> 90%) were determined by RP-HPLC recording the absorbance at 214 nm. To obtain fluorophore-labeled peptides, 5(6)-carboxyfluorescein was coupled to the N-terminus of Onc112, Chex1Arg20, Chex1Arg20 D4K and Apidaecin 1b or at the δ-amino group of ornithine of Api88, Api137 and Api795 upon completion of the peptide synthesis.

### 2.3 Microorganisms, media, and growth conditions

The encapsulated strain *C. neoformans* 1841 (serotype D), which was originally isolated from the cerebrospinal fluid (CSF) of an AIDS patient was obtained from F. Hoffmann-La Roche Ltd., Basel (Decken et al., 1998). H99 and KN99α (both serotype A) were kindly provided by K.J. Kwon-Chung (NIH, Bethesda, MD, USA) and G. Jarbon (Institute Pasteur, Paris, France), respectively.

The strains were grown in Sabouraud medium (SAB, 2% glucose, 1% peptone) overnight at 30°C on an orbital shaker (80 rpm). The cells were centrifuged (10 min, 4°C, 400 × g, Allegra X-22R, Rotor SX4250, Beckmann Coulter, Krefeld, Germany) and the cell pellet was washed with phosphate buffer (10 mmol/L Na_2_HPO_4_/NaH_2_PO_4_, pH 7.5). After a second centrifugation step, the cells were re-suspended and cell counts (mentioned in the respective method section) were adjusted with 50% SAB in phosphate buffer. Colony-forming units (CFU) were determined on SAB agar plates (2% glucose, 1% peptone, 2% agar).

### 2.4 Broth microdilution assay

MIC values were determined using a liquid broth microdilution assay in sterile 96-well plates (polystyrene F-bottom, Greiner Bio-One GmbH) with a total volume of 100 μL per well. Aqueous peptide solutions (3 g/L) were serially diluted twofold with 50% SAB in phosphate buffer starting at a peptide concentration of 128 mg/L (50 μL/well). Overnight cultures were performed as described above. The cell count was adjusted to 1 × 10^5^ CFU/mL to achieve a final concentration of 5 × 10^4^ CFU/mL per well (50 μL/well). Plates were incubated at 30°C and the optical density was measured at 595 nm (OD_595_) after 24 h and 48 h, respectively, using a microplate reader (Victor^3^, Perkin Elmer, Waltham, USA). The MIC was defined as the lowest peptide concentration preventing visible fungal growth. Experiments were performed as triplicates and repeated at least once on another day.

### 2.5 Antifungal activity assay

To determine antifungal activity by plating out on agar plates, overnight cultures were diluted to 5 × 10^4^ CFU/mL with 50% SAB in phosphate buffer. Aqueous peptide solutions were added at various concentrations (20 μL) to the cell suspension (80 μL). Samples were incubated at 30°C on an orbital shaker (750 rpm, Titramax 1,000, Heidolph Instruments GmbH & Co. KG, Schwabach, Germany) for 3 h. After incubation, samples were stored on ice and diluted 30-fold with phosphate buffer. Aliquots of the diluted cell suspensions (100 μL) were plated onto SAB agar plates. Plates were incubated at 30°C and colonies were counted after 24 h and 48 h, respectively.

### 2.6 Time-kill assay

The kinetic effects of the antifungal activity were determined by diluting overnight cultures to 5 × 10^4^ CFU/mL with 50% SAB in phosphate buffer. Aqueous peptide solutions (20 μL) were added to the cell suspension (80 μL) to give a final peptide concentration of 2.5 μmol/L. Samples were incubated at 30°C on an orbital shaker (750 rpm, Titramax 1,000, Heidolph Instruments GmbH & Co. KG) for 24 h. Before incubation (0 min) and after 45 min, 90 min, 3 h, 6 h, and 24 h incubation, aliquots (10 μL) of samples were taken and diluted with phosphate buffer (10 mmol/L, pH 7.5) to observe countable cell colonies. Aliquots (100 μL) of the diluted cell suspensions were plated on SAB agar plates. Plates were incubated at 30°C and colonies were counted after 24 h.

### 2.7 Confocal laser scanning microscopy (CLSM) and deconvolution

Overnight cultures of *C. neoformans* 1841 were prepared as described above and adjusted with 50% SAB in phosphate buffer to reach 5 × 10^6^ CFU/mL. Aqueous peptide solutions (20 μL) were added to the cell suspension (80 μL) to give a final peptide concentration of 25 μmol/L in each well. Samples were incubated at 30°C on an orbital shaker (750 rpm, Titramax 1,000, Heidolph Instruments GmbH & Co. KG) for 3 h. Afterwards, samples were centrifuged (2,000 × g, 10 min, 4°C, Microfuge 22R, Beckmann Coulter), cell pellets were washed with ice-cold phosphate-buffered saline (PBS, 100 μL) and centrifuged again. The supernatant was discarded and the cells were fixed with 4% paraformaldehyde in PBS (25 μL, 30 min, RT). After centrifugation, the cell pellets were re-suspended in 2.5 μL PBS. For immobilization, glycerol-gelatin mounting medium was heated to 60°C in a water bath. A small drop was placed on a microscope slide, the re-suspended cell suspension was pipetted into the drop and covered with a coverslip. Samples were analyzed with a Leica TCS SP8 DMi8 confocal laser-scanning microscope (Leica Microsystems, Mannheim, Germany) using the objective HC PL APO CS2 63x/1.30 GLYC and the software Leica Application Suite X (LAS-X 3.5.7). 5(6)-carboxyfluorescein labeled peptides were excited at 496 nm (HyD detection range 503–600 nm). Measurements across z-axis with 50 to 90 layers (12 to15 μm) were taken.

CLSM images were deconvoluted using the software Huygens Professional 20.10 (SVI, Hilversum, The Netherlands) with following deconvolution parameters: Classic Maximum Likelihood Estimation (CMLE) algorithm, maximum iterations = 30, quality change threshold = 0.01, signal-to-noise-ratio = 13, background = 0. Imaris 9.9 (Oxford Instruments, Abingdon, UK) was used for 3D visualization.

### 2.8 Scanning electron microscopy (SEM)

Overnight cultures of *C. neoformans* 1841 were prepared as described and adjusted with 50% SAB in phosphate buffer to reach 1 × 10^8^ CFU/mL. Aqueous peptide solutions (20 μL) were added to the cell suspension (80 μL) to give a final peptide concentration of 200 μmol/L. Samples were incubated at 30°C on an orbital shaker (750 rpm) for 3 h. Samples were centrifuged (2,000 × g, 10 min, 4°C, Microfuge 22R, Beckmann Coulter), cell pellets were washed with ice-cold phosphate-buffered saline (PBS, 100 μL) and centrifuged again. Cell pellets were re-suspended in PBS (25 μL) and adhered overnight in humid atmosphere to glass coverslips (Borosilicate Glass, VWR International GmbH) previously coated with 0.001% poly-L-lysine (in case of Critical-Point Drying). Cells were fixed (2% glutaraldehyde, 2% paraformaldehyde, in PBS) for 60 min and afterwards stained with 1% OsO4 in PBS for 60 min. The cells were gradually dehydrated in ethanol series (30, 50, 70, 85, 90, 96% (once), and 100% (thrice) for 10 min. The coverslips were critical-point-dried (Baltec CPD 030, BAL-TEC GmbH, Schalksmühle, Germany) or air-dried after incubation with HMDS (3 min, RT) and sputter coated with 20 nm gold/palladium (Baltec MED 020, BAL-TEC GmbH). Cells were visualized using the SEM secondary electron detector (Zeiss EVO LS 15 LaB6, Carl Zeiss Microscopy Deutschland GmbH).

GraphPad Prism 10.0.3 software was used for statistical analysis of the recorded data. A Dunnett’s multiple comparisons test (Ordinary-one-way ANOVA) was performed.

### 2.9 Cytotoxicity

Human embryonic kidney (HEK293) and human hepatoma (HepG2) cells were cultured in Dulbecco’s modified Eagle’s/Ham’s F-12 medium (DMEM/F-12) containing 10% (v/v) fetal bovine serum and 1% (v/v) penicillin/streptomycin. Cells (20,000 in 200 μL per well) were seeded into a 96-well plate (polystyrene F-bottom, Greiner Bio-One GmbH) and incubated for 24 h (37°C, 5% CO_2_). Cells were washed with PBS (100 μL) and peptide solutions (0.6 g/L in DMEM/F-12) were added. The positive control was a dilution series from 12 to 1.5% (v/v) DMSO and the negative control was 12% (v/v) PBS. After incubation (37°C, 24 h, 5% CO_2_), the supernatant was discarded, fresh medium (90 μL/well) and MTT (10 μL/well, 5 g/L in PBS) were added, and the plate was incubated for 4 h (37°C, 5% CO_2_). A solution (100 μL) of sodium dodecyl sulfate (10% (v/v)) in hydrochloric acid (10 mmol/L) was added and the plate was incubated again for 24 h (37°C, 5% CO_2_). Absorbance was recorded at 570 nm relative to the reference at 650 nm (PARADIGM™ microplate reader). All samples were corrected for the background absorbance of the medium. Relative cell viability was calculated as the ratio of absorbances recorded for treated and untreated cells (Kolano et al., 2021; Brakel et al., 2022a,b).

### 2.10 Hemolytic activity

Porcine blood was collected in EDTA tubes, stored at room temperature (RT) for 60 min, centrifuged (5 min, 1,000 × g, 4°C), and the plasma supernatant removed. Concentrated porcine erythrocytes were diluted in PBS (2%) and 50 μL/well were added to a serial peptide dilution series from 600 to 5 mg/L in PBS (50 μL/well) in 96-well polypropylene plates (V-bottom, Greiner Bio-One GmbH) and incubated (37°C, 1 h). The plates were centrifuged (5 min, 1,000 × g, 4°C), the supernatants (100 μL) were transferred to flat-bottom 96-well plates, and the absorbance was recorded at 405 nm on a microplate reader (PARADIGM™, Molecular Devices). PBS was used as negative control, and a serial dilution of triton X-100 from 0.1 to 0.00078% was used as a positive control (Czihal et al., 2012; Mohammed et al., 2023).

## 3 Results

### 3.1 Antifungal activity

**Figure 1:**
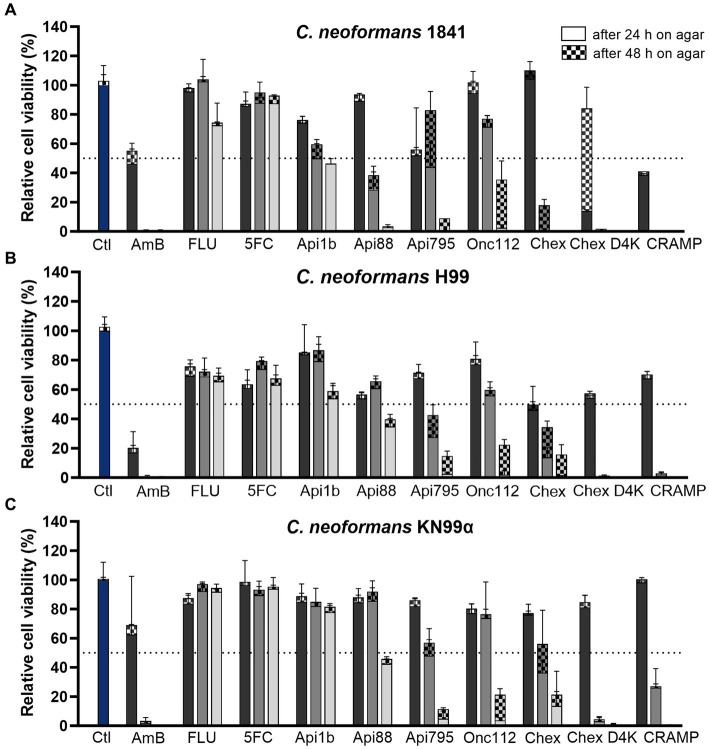
PrAMPs and the cathelicidin CRAMP have a concentration-dependent effect on cell viability and are able to reduce colony-forming units. Relative cell viability of *C. neoformans* strains **(A)** 1841, **(B)** H99, and **(C)** KN99α was assessed after incubation (3 h, 30°C) with AMPs and antifungals at concentrations of 0.25 μmol/L (darker gray), 2.5 μmol/L (gray), and 25 μmol/L (lighter gray). Divided bars show colony-forming units (CFU) counted after 24 h (no pattern) and 48 h (checkered) incubation at 30°C. Samples were normalized to the control sample (no peptide or antifungal, blue). Dotted line indicates 50% inhibition. Shown is the mean of four replicates (*n* = 4) performed as duplicates on two separate days and the associated standard deviation. Absolute CFU counts are shown in Supplementary Figure S1 and Supplementary Table S2. Ctl – control; AmB – Amphotericin B; FLU – fluconazole; 5FC – flucytosine; Chex – Chex1Arg20; Chex D4K – Chex1Arg20 D4K.

Since *C. neoformans* infections can be caused by different serotypes (A and D) and mating types (a and α), three strains were included in this study. To investigate the influence of serotype on AMPs activity on fungi, strain *C. neoformans* H99 was chosen as an example for serotype A and *C. neoformans* 1841 for serotype D. The sexual morph KN99α, which is closely related to strain H99, was included because the mating type α (MATα) form is the more virulent form (Lin and Heitman, 2006). Thus, possible influences of the differences in the strains on the activity can be detected. The activity of 20 AMPs from four different PrAMP families, CRAMP, reverse CRAMP (sequences listed in Table 1), and the antifungals amphotericin B, fluconazole, and flucytosine were tested against *C. neoformans* strains 1841 (serotype D), H99 (serotype A), and KN99α (serotype A, mating type MATα) using a microdilution assay in 96-well-plates (Table 2). Although clear and reproducible MICs were already obtained after 24 h of incubation, the plates were further incubated and second MICs were determined after a total incubation time of 48 h. Interestingly, significant differences were observed among the PrAMPs, despite similar sequence motifs consisting of proline and the basic amino acids arginine, lysine, and ornithine. Natural insect-derived PrAMPs, i.e., apidaecin 1b, drosocin, and pyrrhocoricin, were inactive against *C. neoformans*, i.e., MICs of > 61, 58, and 55 μmol/L (always > 128 mg/L), respectively, whereas analogs optimized for enhanced antibacterial activities, such as Api795, Api813, Api822, Onc112, and Chex1Arg20, showed moderate to good activities with MICs ranging from ~1.6 to 6.7 μmol/L (i.e., 4 to 16 mg/L). Interestingly, all three *C. neoformans* strains tested were equally susceptible to each peptide, e.g., the MIC of the most active peptide Chex1Arg20 D4K was always 1.6 μmol/L (4 mg/L). Amphotericin B was highly active (< 0.22 μmol/L; < 0.2 mg/L), fluconazole was moderately active with strain-dependent MICs ranging from 13 to 105 μmol/L (4 to 32 mg/L), and flucytosine was virtually inactive with MICs of at least 496 μmol/L (64 mg/L) under the conditions tested. In addition, the CFU counts were determined after incubating *C. neoformans* strains with selected peptides or antifungal agents for three hours before plating the cell suspensions on agar (Figure 1). The lowest concentration of PrAMPs tested (0.25 μmol/L) reduced the CFU by ~24% (± 15%) on average after 48 h. At 10-fold higher concentrations (2.5 μmol/L), the CFU varied much more for the different peptides. For example, Api88 did not significantly reduce the CFU of *C. neoformans* strains H99 and KN99α, whereas CRAMP and Chex1Arg20 D4K strongly reduced the CFU with only a few colonies observed. With the exception of apidaecin 1b, fungal cell viability was reduced by an average of 81% by all peptides at the highest peptide concentration tested (25 μmol/L). Chex1Arg20 D4K and CRAMP completely suppressed the fungi at peptide concentrations ≥2.5 μmol/L, with no colonies visible even after 48 h. Interestingly, delayed colony formation was observed for Chex1Arg20, Onc112, and Api795, with only small colonies appearing after more than 24 h.

**Table 1:** List of peptides used, including their sequence, number of amino acids (aa), molecular weight (MW), and net charge.

Peptide	Sequence	Length (aa)	MW (g/mol)	Net charge
**Api88**	gu-ONNRPVYIPRPRPPHPRL-NH_2_	18	2,289	+6
**Api88 rev**	gu-LRPHPPRPRPIYVPRNNO-NH_2_	18	2,289	+6
Api88 rev*	gu-LRPHPPRPRPRPIYVPRNNO-NH_2_	20	2,542	+7
**Api88 scr**	gu-OPNRYIRPRLPPHPNRPV-NH_2_	18	2,289	+6
**Api137**	gu-ONNRPVYIPRPRPPHPRL-OH	18	2,290	+5
**Api795**	gu-OIOIORPVYOPRPRPPHPRL-OH	20	2,517	+7
**Api813**	gu-OIOIORPVYOPRPRPPHPRR-OH	20	2,560	+9
**Api822**	OIOIORPVYOPRPRPPHPRR-OH	20	2,463	+9
**Apidaecin 1b**	GNNRPVYIPQPRPPHPRL-OH	18	2,107	+3
**Apidaecin 1b scr**	GPLRIYVPHPPPRPNQNR-OH	18	2,107	+3
**Onc72**	VDKPPYLPRPRPPROIYNO-NH_2_	19	2,304	+6
**Onc72 rev**	ONYIORPPRPRPLYPPKDV-NH_2_	19	2,304	+6
**Onc112**	VDKPPYLPRPRPPRrIYNr-NH_2_	19	2,388	+6
**Chex1Arg20**	ChexRPDKPRPYLPRPRPPRPVR-NH_2_	20	2,475	+7
**Chex1Arg20 rev**	RVPRPPRPRPLYPRPKDPRChex-NH_2_	20	2,475	+7
**Chex1Arg20 scr**	DRVRPRPKPRChexRPPYRPPLP-NH_2_	20	2,475	+7
**Chex1Arg20 D4K**	ChexRPKKPRPYLPRPRPPRPVR-NH_2_	20	2,488	+9
**Drosocin**	GKPRPYSPRPTSHPRPIRV-OH	19	2,199	+5
**Pyrrhocoricin**	VDKGSYLPRPTPPRPIYNRN-OH	20	2,340	+3
**Bac7 1–60**	RRIRPRPPRLPRPRPRPLPFPRPGPRPIPRPLPFPRPGPRPIPRPLPFPRPGPRPIPRP-OH	60	7,023	+17
**CRAMP**	GLLRKGGEKIGEKLKKIGQKIKNFFQKLVPQPEQ-OH	34	3,876	+6
**CRAMP rev**	QEPQPVLKQFFNKIKQGIKKLKEGIKEGGKRLLG-OH	34	3,876	+6

rev-reversed sequence; scr – scrambled sequence; gu − *1,1,3,3* tetramethyl guanidine; NH_2_ – C-terminal amidation; Chex – 1-amino cyclohexyl carboxylic acid; O – l-ornithine; r – d-arginine.

**Table 2:** Minimum inhibitory concentrations (MICs) of tested peptides are sequence-dependent. MICs were determined for various peptides and antimycotics against the *C. neoformans* strains 1841, H99, and KN99α.

	**MIC (μmol/L)**					
	**1841**		**H99**		**KN99α**	
**Peptide**	**24 h**	**48 h**	**24 h**	**48 h**	**24 h**	**48 h**
**Api88**	3.5	7.0	7.0	3.5	3.5	7.0
**Api88 rev**	7.0	7.0	7.0	14	7.0	14
Api88 rev*	3.1	3.1	3.1	3.1	3.1	3.1
**Api88 scr**	7.0	14–28	28	56	28	56
**Api137**	14	28	14	28	14–28	28–56
**Api795**	1.6	3.2	3.2	3.2	1.6–3.2	3.2
**Api813**	3.1	3.1	1.6	1.6–3.2	1.6–3.2	1.6–3.2
**Api822**	3.2	3.2	3.2	3.2	3.2	3.2
**Apidaecin 1b**	> 61	> 61	> 61	> 61	> 61	> 61
**Apidaecin 1b scr**	> 61	> 61	> 61	> 61	> 61	> 61
**Onc72**	28	56	14	28–56	14–28	56
**Onc72 rev**	28	56	28	56	28	56
**Onc112**	3.3	6.7	3.3–6.7	6.7	3.3–6.7	6.7–13
**Chex1Arg20**	1.6	3.2	1.6	1.6–3.2	1.6	3.2
**Chex1Arg20 rev**	3.2	3.2	3.2	3.2–6.5	3.2	6.5
**Chex1Arg20 scr**	13–26	26–52	26	52	26	52
**Chex1Arg20 D4K**	1.6	1.6	1.6	1.6	1.6	1.6
**Drosocin**	29	58	58	>58	>58	>58
**Pyrrhocoricin**	>55	>55	>55	>55	>55	>55
**Bac7 1–60**	1.1	1.1	1.1	1.1	1.1	1.1–2.2
**CRAMP**	2.1	2.1	2.1	2.1	2.1	2.1
**CRAMP rev**	1.0	2.1	1.0	2.1	1.0–2.1	2.1
**Amphotericin B**	0.06–0.23	0.13	<0.02	0.04–0.09	0.09	0.17
**Fluconazole**	105	105	53	53–105	13	26
**Flucytosine**	496	992	496	>992	992	>992

The MIC values were determined twice at 24 h and 48 h. Shown is the mean of six replicates (*n* = 6) performed as triplicates on two separate days. MICs are expressed in μmol/L. MICs expressed in mg/L are listed additionally in Supplementary Table S1. * at Api88 rev* indicates an extended version with a sequence listed in Table 1.

Both the MICs and the CFUs indicated that the peptide charge might influence their antifungal activity. Apidaecin 1b, with its moderate positive net charge of +3, was inactive against *C. neoformans* (MIC > 61 μmol/L; > 128 mg/L), whereas the related peptides Api137 and Api88, with higher net charges of +5 and + 6, respectively, showed enhanced activity. Api137 showed a moderate improvement of antifungal activity with MICs of 14 to 28 μmol/L (i.e., 32 to 64 mg/L). Amidation at the C-terminus (Api88) further improved the MIC to as low as 3.5 μmol/L (8 mg/L). Similarly, substitution of Asp4Lys in Chex1Arg20 improved antifungal properties of Chex1Arg20 D4K. Although the MIC decreased by only one dilution step from 3.2 to 1.6 μmol/L (8 to 4 mg/L), this substitution prevented the “re-growth” effect on agar plates observed for Chex1Arg20. Unexpectedly, substitution of d-Arg in Onc112 to Orn (Onc72) increased the MIC by up to three dilution steps, despite similar net charges at neutral pH.

**Figure 2:**
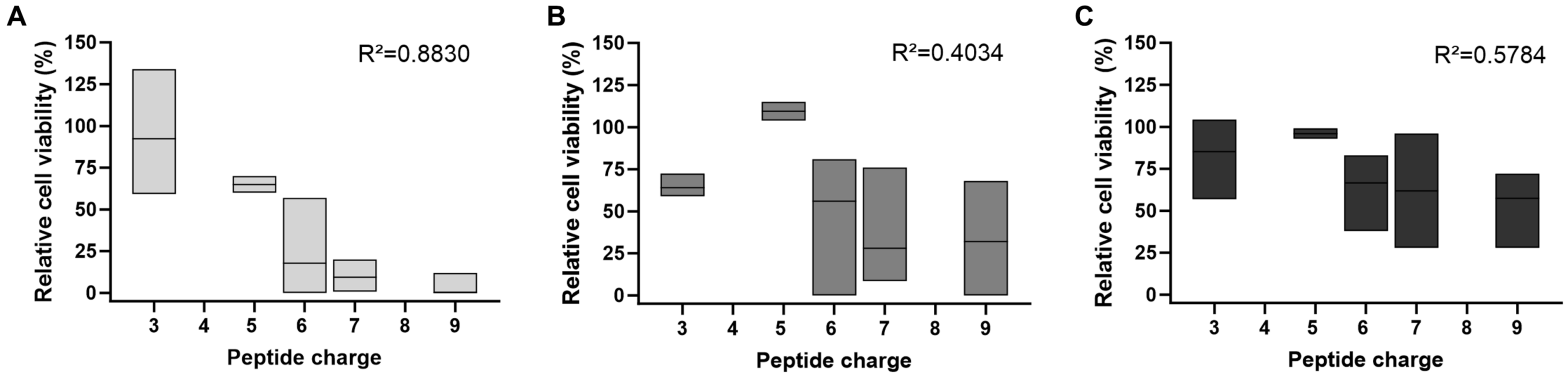
The peptide net charge influences the antifungal activity against *C. neoformans* 1841, which depends on the peptide concentration used. Antifungal activity was determined by the number of CFU on SAB agar after incubation with **(A)** 25 μmol/L, **(B)** 2.5 μmol/L, or **(C)** 0.25 μmol/L peptide, shown as floating bar graph. Results were normalized to the control sample (no peptide). CFU counts were determined after 48 h. Data rely on all peptides listed in Supplementary Table S1, except Bac7 1–60 as well as the reverse and scrambled sequences. The mean between peptides is indicated with a solid line. Simple linear regression tests (Pearson correlation) were performed using GraphPad Prism 10.0.3.

The trend of a more pronounced effect with higher peptide concentrations was particularly evident in the determination of CFU after plating cell suspensions incubated with 25 μmol/L peptide (Figure 2A). In this case, increased net charges correlated well with decreased cell viability (R^2^ = 0.8830). This trend was also observed at 10- and 100-fold lower peptide concentrations, although it was not significant (R^2^ = 0.4034 and R^2^ = 0.5784, Figures 2B,C).

**Figure 3:**
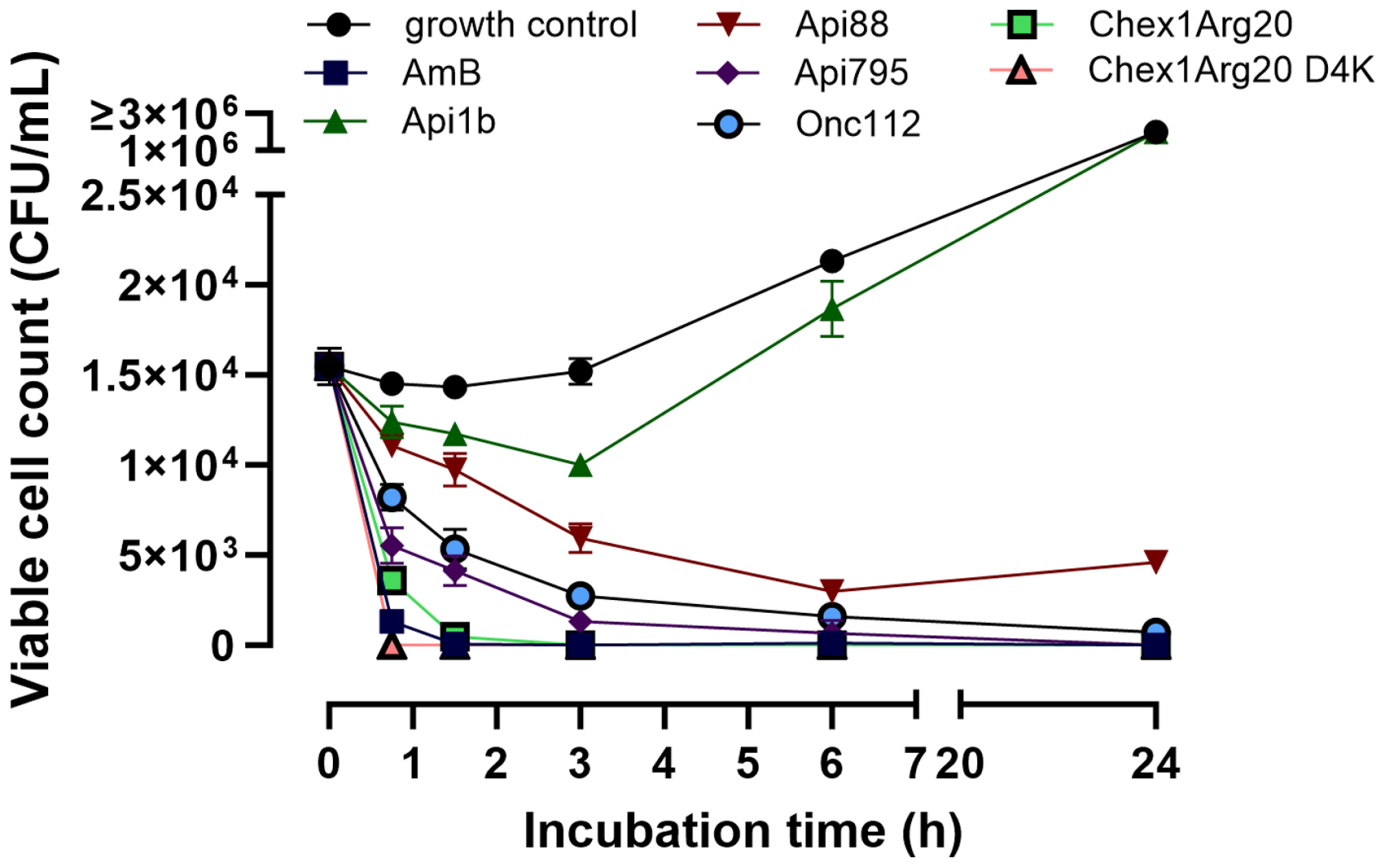
PrAMPs show a time-dependent effect on *C. neoformans* 1841. Fungal cells were incubated with peptides or Amphotericin B (2.5 μmol/L) and aliquots were plated after defined time point for determination of the CFU. An untreated sample is used as a growth control. The mean value from triplicates with the corresponding standard deviation is shown.

A time-kill assay showed that in addition to different dose-dependent activities, the peptides also had time-dependent effects. Therefore, the fungal cells were incubated with peptides at concentrations close to the MIC values of the active peptides (2.5 μmol/L) and aliquots were plated after defined time points to determine the CFUs. No viable cells were detected after 45 min of incubation with Chex1Arg20 D4K (Figure 3), which was only slightly slower than amphotericin B. The PrAMPs Api88, Onc112, and Api795 showed a delayed antifungal effect, but showed a clear effect after 3 h. Apidaecin 1b had only a weak effect in the first 3 h before the CFU increased again to the CFU of the control.

To gain insight into the mechanism, reverse sequences of selected peptides were tested. For this purpose, one active peptide from the apidaecin (Api88) and oncocin groups (Onc72), Chex1Arg20, and CRAMP were selected and synthesized with a reverse sequence. Unexpectedly, all reverse peptides were as active as the corresponding forward sequences. Therefore, the highly active peptides Api88 and Chex1Arg20, which have been extensively studied in our group, and apidaecin 1b (control) were selected to synthesize peptides with the scrambled sequences. All three scrambled peptides were basically inactive, especially considering the MICs at 48 h, which increased from 3.6 to 52 μmol/L (8 to 64 or 128 mg/L) for Chex1Arg20 and from 7.0 to 28 or 56 μmol/L (16 to 64–128 mg/L) for Api88 (Table 2). The scrambled sequence of apidaecin 1b remained inactive. This clearly showed that the activity was sequence-dependent, with forward and reverse sequences showing equal activity.

### 3.2 Confocal laser scanning microscopy (CLSM)

CLSM was used to investigate whether the PrAMPs studied enter fungal cells in a manner similar to bacterial cells (Mattiuzzo et al., 2007; Krizsan et al., 2015a), as opposed to mammalian cells (Hansen et al., 2012; Bluhm et al., 2016). This technique allows investigating whether the peptides are mainly present in the capsule and membrane region, or whether they are able to penetrate the capsule and cell membrane and reach the cytoplasm. Thus, N-terminally 5(6)-carboxyfluorescein (*Cf*)-labeled peptides Onc112, Chex1Arg20 (and D4K), and apidaecin 1b as well as Api88, Api137 and Api795 carrying *Cf* at the δ-amino group of Orn-1 due to guanidation of the N-terminus were synthesized. *Cf*-Api795, *Cf*-Onc112, and *Cf*-Chex1Arg20 were slightly more active against *C. neoformans* 1841, while *Cf*-Chex1Arg20 D4K, *Cf*-Api88, and *Cf*-Api137 were slightly less active than the corresponding unlabeled peptides (Supplementary Table S3). Since all *Cf*-labeled peptides were similarly active as the unlabeled analogs on *C. neoformans* 1841, we assumed that their cellular distribution is also very similar. Thus, fungal cultures were incubated with no peptide (control) or one of the *Cf*-labeled peptides, which were selected from different peptide families with different antifungal activity, at 30°C for 3 h. The cells were prepared for CLSM, immobilized on glass slides, and studied under the microscope using light imaging and fluorescence.

**Figure 4:**
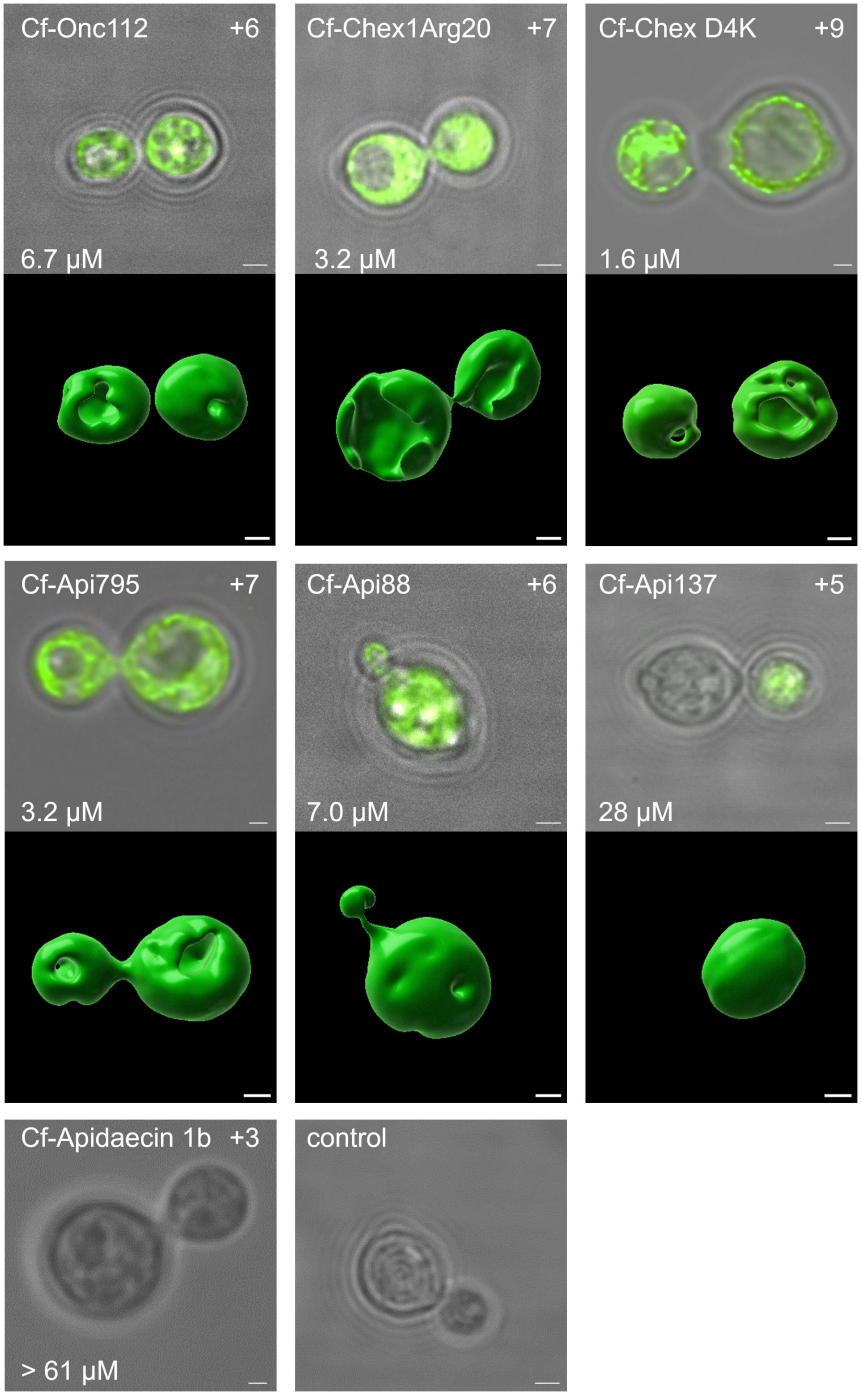
The antifungal activity as well as the net charge of the peptides was found to correlate with the intracellular uptake. Confocal laser scanning images of *C. neoformans* 1841 cells incubated with *Cf*-labeled peptides (25 μmol/L, 30°C, 3 h). Cells were immobilized on glass slides and the fluorescence was measured for the entire cell (λ_ex_ = 496 nm, λ_em_ = 503–600 nm). For each peptide, the central z-stack was shown as an overlay of the white light image and fluorescence channel after deconvolution. The MIC (after 48 h, lower left corner) and net charge (upper right corner) are provided for each peptide as well. Additionally, for peptides with detectable fluorescence, a 3D visualization was prepared using Imaris. Scale bar, 1 μm.

As expected, the control sample showed no fluorescence, whereas cells incubated with *Cf*-labeled sequences of Onc112, Chex1Arg20, Chex1Arg20 D4K, Api795, and Api88 showed intense, non-uniformly distributed fluorescence, mainly in the cytoplasmic region. Whole-cell scans clearly demonstrated the fluorescence inside the cells, although some cells showed more intense spots for *Cf*-Api88 or less fluorescent areas for *Cf*-Api795, *Cf*-Chex1Arg20 D4K, and *Cf*-Onc112 (Figure 4; Supplementary Figure S3). These observations could not be attributed to distinct cellular compartments. To gain a more comprehensive understanding of the peptide distribution within the cells, a 3D visualization was conducted. This analysis unveiled certain regions within the cells where no fluorescence signal was detected, indicating the absence of the peptide in these areas. However, it remained open, whether this lack of fluorescence is due to a dented or damaged membrane.

In some cells near the daughter cell encapsulation, the *Cf*-labeled peptide appeared to be transferred from the mother cell to the daughter cell. Interestingly, mother and daughter cells often showed similar fluorescence intensities (Figure 4, *Cf*-Chex1Arg20, *Cf*-Api795), but sometimes one of the cells was less fluorescent (Figure 4 *Cf*-Api137, Supplementary Figure S2 *Cf*-Api88, *Cf*-Onc112). In some images, two cells appeared to “exchange” the fluorescence (Supplementary Figure S2 *Cf*-Onc112, *Cf*-Api795, Figure 4 *Cf*-Chex1Arg20, *Cf*-Api795). Whether the cells were already within the budding process at the time of peptide addition and the peptides were taken up simultaneously by mother and daughter cell or whether the cell only transferred the peptide from mother to daughter cell during early budding after peptide uptake is not evident from this experiment.

Fungi incubated with *Cf*-Api137 showed much weaker or no fluorescence, requiring much higher laser power to excite the fluorophore, most likely indicating a much lower concentration than observed for *Cf*-Api88. This was even more evident with the inactive sequence *Cf*-apidaecin 1b, where no fluorescence could be detected in the majority of cells. However, a few cells displayed a fluorescence in the cytosol (Figure 4; Supplementary Figure S3). In general, the antimicrobial activity correlated with the fluorescence intensity and thus with the presence of the peptide in the cytoplasm.

### 3.3 Scanning electron microscopy

**Figure 5:**
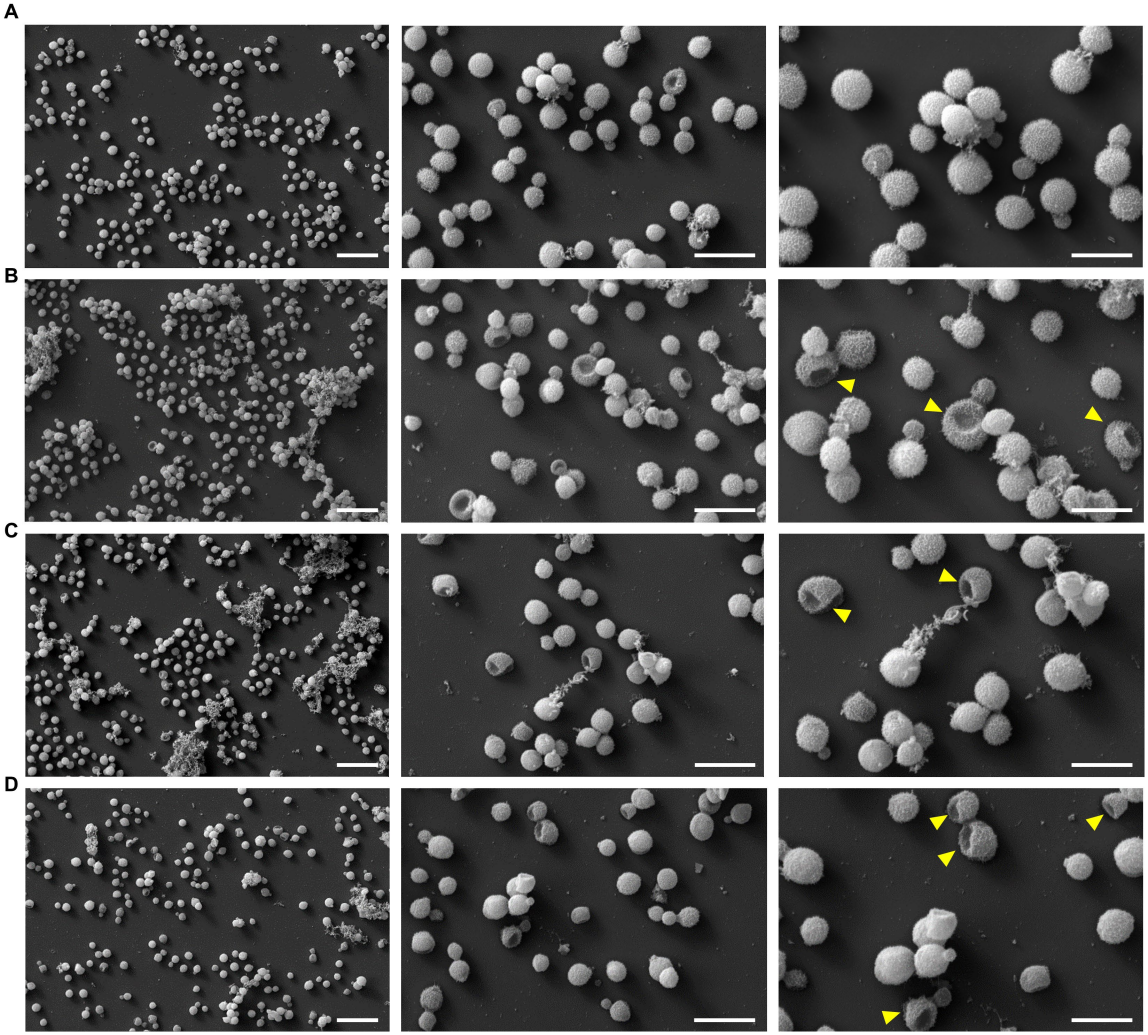
Images of scanning electron microscopy showed damaging effect on fungal cell exterior. Cells were incubated **(A)** without peptide (control) or with **(B)** Chex1Arg20, **(C)** Api88 or **(D)** Onc112 and visualized using three different magnifications [5 k (left, scale bar 20 μm), 15 k (middle, scale bar 10 μm), and 25 k (right, scale bar 6 μm)]. Examples of damaged fungal cells are marked with a yellow arrow in the 25 k figures.

**Figure 6:**
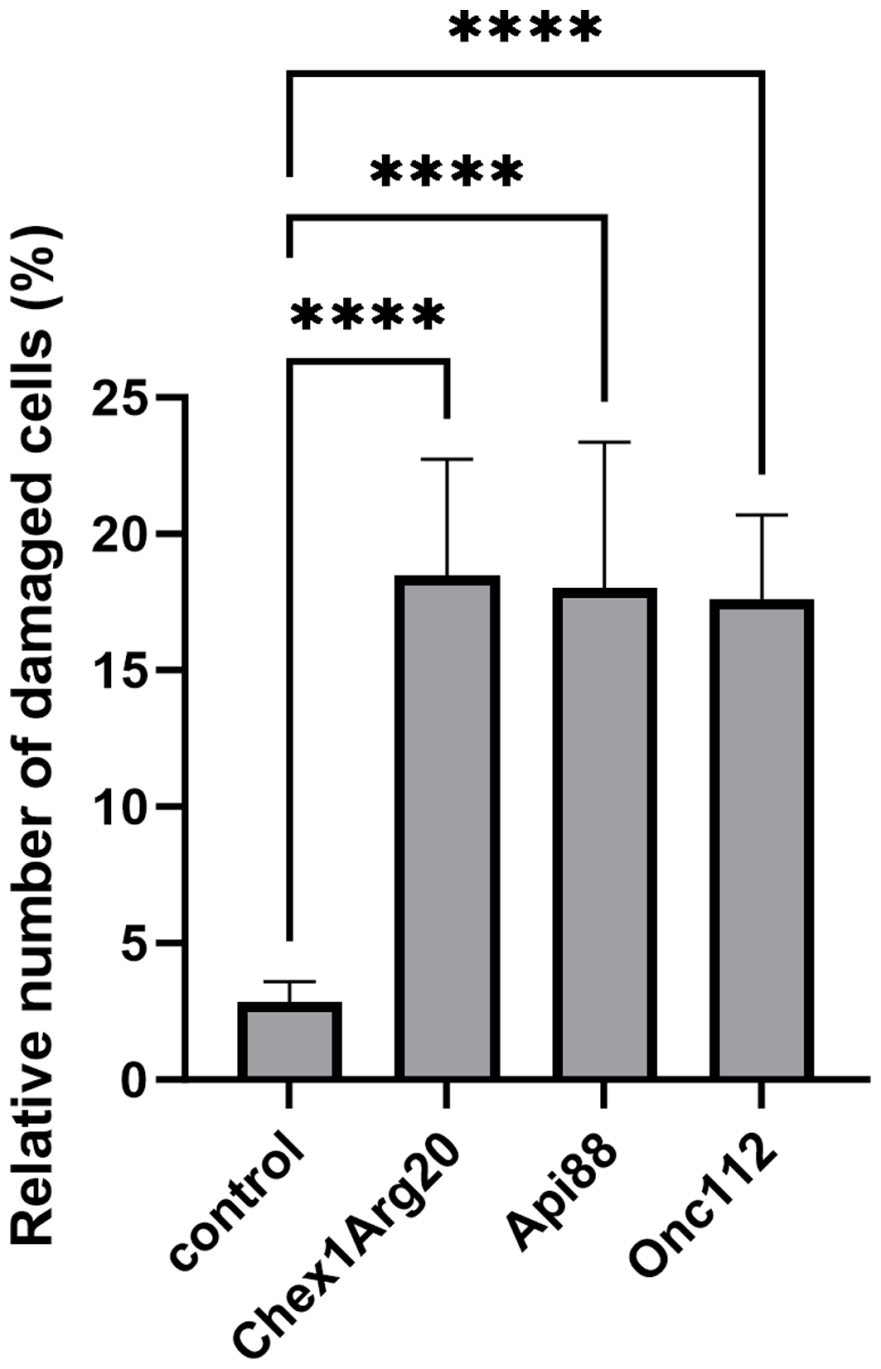
Fungal cells treated with the PrAMPs Chex1Arg20, Api88, and Onc112 showed partially indentation. Cells were incubated without peptide (control) or with Chex1Arg20, Api88 or Onc112. All samples shown were treated under the same conditions. Damaged and intact cells from five SEM images were counted per peptide, and the relative proportion of damaged cells was calculated. Data represent the mean including the standard deviation (*n* = 5). Data were analyzed using Dunnett’s multiple comparisons test (GraphPad Prism 10.0.3, **** *p* < 0.0001).

To test possible lytic effects of the peptides on the fungi, the fungi were treated with PrAMPs and examined by SEM (Figure 5). The cell number and corresponding peptide concentration had to be increased due to the more complex sample preparation compared to the antifungal activity assay and CLSM. A control sample treated in the same manner but without the addition of peptide predominantly showed intact, round cells with only ~1 to 3% of the cells damaged. When incubated with PrAMPs Chex1Arg20, Api88, and Onc112 the majority of cells appeared intact, only a small portion showed deep indentations (Figures 5, 6). No significant differences were observed between the three peptides. Although very low, the proportion of damaged cells within the peptide-treated cells was significantly higher than in the untreated control cells. In order to confirm these observations and to analyze whether other PrAMPs, including the inactive peptide apidaecin 1b, induce a similar effect on the fungi, cells were treated with seven different PrAMPs in a second batch (Supplementary Figure S4). Cells with comparable damage were observed for all peptides with the proportion of damaged cells being significantly higher in all peptide-treated cells than in the control (Supplementary Figure S4). The lowest percentage of damaged cells was observed for apidaecin 1b. Similarly, treatment with the less active peptide Chex1Arg20 scrambled resulted in a slightly lower percentage of damaged cells than Chex1Arg20, although no significant difference was observed between the two peptides. Similarly, there were no significant differences between cells treated with Chex1Arg20 and the reverse sequence or the derivative with the D4K substitution. Overall, round shaped cells were observed in the treated samples, with the exception of indentations, while no other damage to the cell capsule was observed in contrast to cells incubated with DMSO (data not shown). Additionally, cells in various stages of budding were observed. Thus, the antifungal activity of the tested peptides did not correlate with the observed rather low damage to the cell capsule, indicating a minor side effect.

### 3.4 Cytotoxicity and hemolytic activity

**Figure 7:**
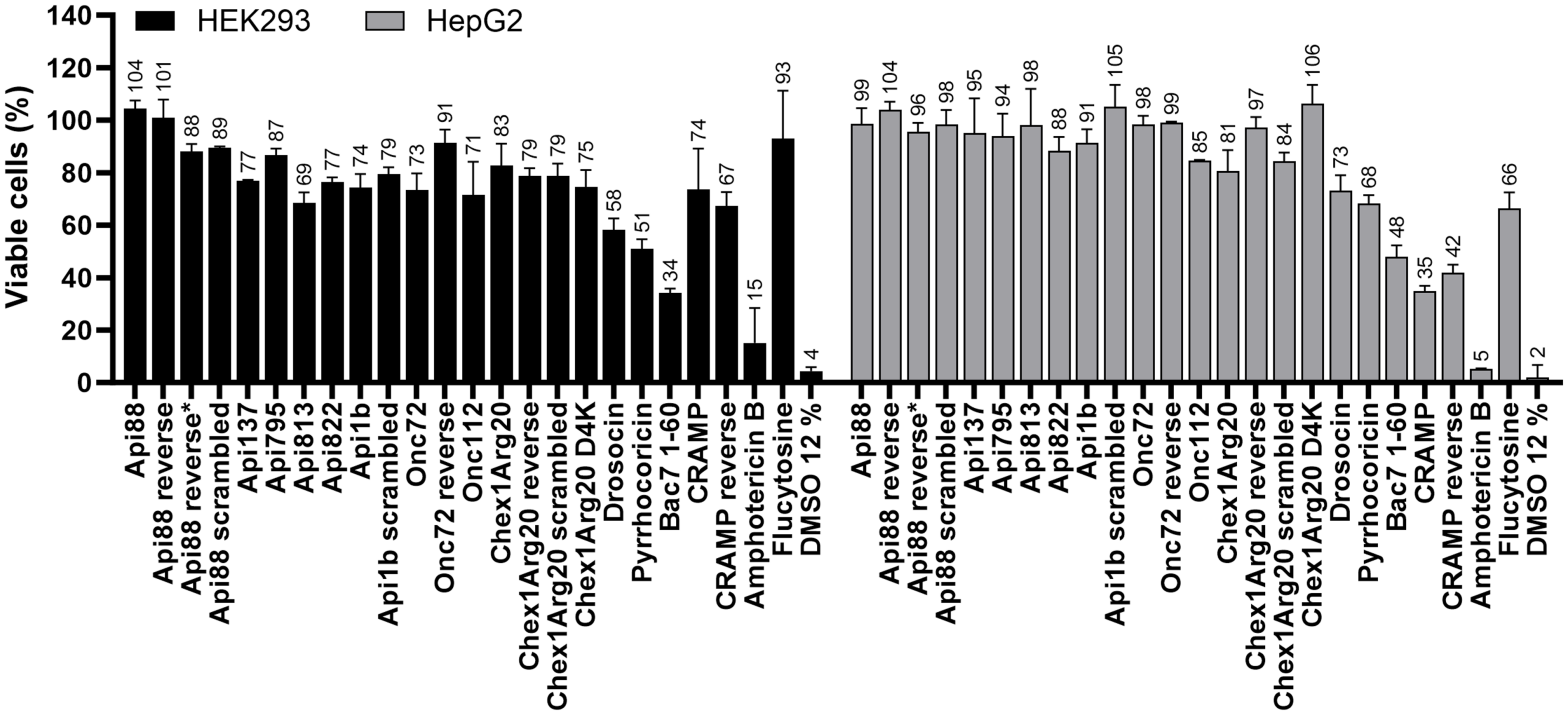
Effect of AMPs and antifungal agents on the cell viability of eukaryotic cell lines HEK293 (black) and HepG2 (grey). Cells were incubated with peptides (0.6 g/L), flucytosine (0.6 g/L) or amphotericin B (0.125 g/L) for 24 h. Samples were normalized to a sample incubated with PBS (12%). A sample incubated with DMSO (12%) served as a positive control. Each bar represents the mean of triplicates with the corresponding standard deviation.

Having observed the high activity and partially rapid kill kinetics on eukaryotic fungal cells, the active peptides including the reverse and scrambled peptides were studied for possible toxic effects on eukaryotic mammalian cells, i.e., human liver (HepG2) and kidney cell lines (HEK293) (Figure 7). In agreement with previous studies (Czihal et al., 2012; Fritsche et al., 2012; Kolano et al., 2021; Brakel et al., 2022a,b), the cell viability of both cell lines was only slightly reduced by the most active peptides at peptide concentrations of 600 mg/L (between 234 and 285 μmol/L). The peptide concentration used was 150-fold above the MIC for the most active peptide (Chex1Arg20 D4K), which demonstrates a high safety margin. Reverse and scrambled sequences also had no cytotoxic effect. In contrast, amphotericin B (125 mg/L) showed strong cytotoxicity, as already described in the literature (Gurudevan et al., 2018).

All PrAMPs tested, including the reverse and scrambled peptides, showed no significant hemolytic activity compared to the untreated control (addition of PBS) up to concentrations of 600 mg/L (234 to 285 μmol/L), as previously reported for some PrAMPs (Czihal et al., 2012; Krieger et al., 2021; Mohammed et al., 2023), in contrast to a strong hemolytic effect observed for amphotericin B already at the lowest concentration tested (125 mg/L; 135 μmol/L) (Supplementary Figure S5).

## 4 Discussion

Cryptococcal meningitis and cryptococcosis caused by *C. neoformans* infections are typically treated with a combination of high-dose liposomal amphotericin B, flucytosine, and fluconazole, followed by a prolonged low-dose fluconazole therapy for at least 8 weeks (Ngan et al., 2022). However, access to liposomal amphotericin B and flucytosine and safe administration are severely limited, especially in developing countries, leading to the use of less effective regimens. Moreover, these drugs have tremendous side effects due to their high toxicity, particularly kidney damage. In general, the development of antifungal drugs is more challenging compared to drugs targeting bacteria, because the membranes and targets of eukaryotic fungal cells are more similar to those of mammalian cells (Denning and Bromley, 2015; Chen et al., 2021). For instance, the peptide indolicidin acts intracellularly against bacteria by inhibiting DNA, RNA, and protein synthesis, but has a membrane-disrupting effect on fungal cells (Subbalakshmi and Sitaram, 1998; Lee et al., 2003). AMPs could be a valid alternative therapeutic strategy, but positively charged AMPs often utilize a membrane-disrupting effect and a lytic mode of action, resulting in an increased risk of cytotoxicity to mammalian cells (Brakel et al., 2022a,b). Research on PrAMPs has mainly focused on their activity against Gram-negative and Gram-positive bacteria and has linked their activity to intracellular targets, i.e., the bacterial 70S ribosome and DnaK, mostly based on research in *Escherichia coli*. PrAMPs are highly efficient in murine models of bacterial infection and show only mild side effects (Knappe et al., 2012). Low cytotoxicity against various mammalian cells, e.g., HEK, Hep and HeLa, and hemolytic activity has already been shown for a variety of PrAMPs (Lai et al., 2019; Kolano et al., 2021; Brakel et al., 2022a,b). This was confirmed in this publication, including reversed and scrambled sequences. In addition, confocal laser scanning microscopy experiments showed that Api137, among others, is not taken up into HeLa cells (Hansen et al., 2012; Bluhm et al., 2016). Similarly, PrAMPs do not have any immunomodulatory effects and do not affect dendritic cells or macrophages (Fritsche et al., 2012). Therefore, they appear to be promising candidates for the treatment of fungal infections. However, the antifungal activity, especially against *C. neoformans*, and the underlying mechanisms are poorly understood. Only two mammalian PrAMPs, Bac7 (1-35) and SP-E, have been shown to be effective against *C. neoformans*, with MIC values as low as 4 μmol/L (Benincasa et al., 2004; Conti et al., 2013). Although intact cell membranes suggested a non-lytic mechanism, the mode of action was not further investigated. Compared to these peptides, the insect-derived PrAMPs studied here are much shorter and have different motifs. Nevertheless, they showed comparable or even superior non-lytic antifungal activity against *C. neoformans*. The most active peptide, Chex1Arg20 D4K, reached an MIC of 1.6 μmol/L (4 mg/L), which is similar to the widely investigated AMPs, such as the cathelicidin CRAMP (2.1 μmol/L, this study), the defensin mimetic brilacidin (2.5 μmol/L, (Dos Reis et al., 2023)), the artificial VG16KRKP (10 μmol/L, (Datta et al., 2016)) or Bac7 1–60 (1.1 μmol/L, this study). Importantly, Chex1Arg20, Api88, and Onc112 showed similar good activities against all three *C. neoformans* strains tested. It is very encouraging that the peptides were equally active against the tested serotype A strains H99 and KN99α (*C. neoformans* var. *grubii*) and serotype D strain 1841 (*C. neoformans var. neoformans*). As mentioned above, the more virulent serotype A is globally dominant, while serotype D prevails in Europe. Therefore, it is crucial for antifungal agents to exhibit activity against both serotypes. Differences between the strains, e.g., capsule composition or melanin production, which may occur, did not appear to affect the antifungal activity (Samarasinghe et al., 2018; Casadevall et al., 2019).

Even though fungal cells and bacterial cells differ in their composition and structure, an overlap in the activity against *C. neoformans* and Gram-negative bacteria was observed for some of the PrAMPs tested. This has the potential advantage of translating previous findings and observations to fungi. The finding that peptides that are active against bacteria *in vitro* are also effective *in vivo*, as has already been shown in various infection models, provides hope for the potential efficacy of PrAMPs *in vivo* against *C. neoformans* (Ostorhazi et al., 2014; Schmidt et al., 2016, 2017). Importantly, these PrAMPs were as efficient as standard antibiotics in these mouse infection models, despite much higher MICs, suggesting that the MICs for bacteria must be evaluated differently than for small molecules. If this is also true for fungi, it would indicate high efficacy against *C. neoformans* infections (Knappe et al., 2015). However, further investigation and confirmation are still required. So far, only *in vitro* data have been considered in this study, which may not accurately reflect the conditions *in vivo*. This has been observed with antifungal agents such as fluconazole and flucytosine, which are effective in treating *C. neoformans* infections but show lower activity under the *in vitro* conditions used. Interestingly, even small structural changes in the apidaecin sequences, i.e., apidaecin 1b, Api88, and Api137, had a strong effect on the antifungal activity, suggesting a sequence-specific target interaction with forward and reversed sequences showing similar activities.

The putative intracellular mode of action is a very significant finding, supported by CLSM images showing fluorescence in the cytoplasmic region after incubation of *C. neoformans* with *Cf*-labeled PrAMPs (Figure 4; Supplementary Figure S3). At this point, we can only speculate about potential targets, such as the ribosomes at the endoplasmic reticulum, which is the primary target of PrAMPs in bacteria, i.e., the 70S ribosome (Krizsan et al., 2014, 2015a,b; Seefeldt et al., 2015; Graf et al., 2017). Binding of PrAMPs to the eukaryotic 80S ribosome has not yet been demonstrated. However, studies have shown that the PrAMP Bac7 (1-35) can inhibit *in vitro* translation in a eukaryotic system, albeit with a 2.5-fold higher IC_50_ compared to the *E. coli* system (Seefeldt et al., 2016). Given the activity of Bac7 (1-35) against *C. neoformans*, one could speculate that the 80S ribosome might be a possible target (Benincasa et al., 2004). Interestingly, the reverse sequences did not seem to have a decisive influence, as similar MIC values were measured for Api88, Onc72, and Chex1Arg20 and the corresponding reverse sequences. In contrast, the sequence was important for the activity of Api88 and Chex1Arg20, as indicated by the high MICs for scrambled sequences, presumably due to poorer target binding (Table 2). Initial investigations using SEM showed that the PrAMPs, may also have a slight membrane-damaging effect on the fungi (Figure 5), as some cells showed a dented shape. Similar membrane damage was already observed by (Sharma et al. (2017, 2022)). However, based on the results shown here, it is unlikely that damage to the cell exterior is the major mechanism of PrAMPs, as it was observed also for inactive sequences, such as Chex1Arg20 scrambled. There was no clear trend between membrane damage and MIC values for the peptides tested, suggesting that the observed cell damage is a minor side effect caused by the sample preparation conditions, probably occurring in cells already killed by the peptides. In addition, peptide concentrations well above the MIC were used due to the relatively high cell number (10^8^ CFU/mL) required for sample preparation. In addition, no nucleotide release from the cytosol could be detected after incubation with the PrAMPs, which makes membrane leakage unlikely. Interestingly, incubation with CRAMP resulted in a slight increase in nucleic acid release (Supplementary Figure S6; Method M1).

Interestingly, the fungi could be observed in different budding states after peptide incubation with CLSM, ranging from cells with early budding to those with almost completed budding (Figure 4). However, since these observations are only snapshots, it remains open whether the fungi were still capable to complete the budding process despite the peptide treatment or whether budding was halted at this stage. Nevertheless, the peptide was distributed in the mother and daughter cells, although partially different fluorescence intensities were observed in each cell. Assuming that the budding process is not stalled by the peptide uptake, the peptide would be distributed to the nascent cells, resulting in a lower (diluted) peptide concentration in each cell. This could explain the observed regrowth effects on agar plates.

The CLSM data also suggest that cellular uptake of *Cf*-Api137 and *Cf*-Apidaecin 1b is less efficient than for *Cf*-labeled Chex1Arg20, Api88, and Onc112, which may potentially explain the lower activity of Api137 and the inactivity of apidaecin 1b. The only difference between Api137 and Api88 is the C-terminus, i.e., a carboxyl group instead of an amide, respectively, which decreases the peptide net charge by one. The importance of the peptide charge has been reported for the uptake of PrAMPs in *E. coli (*Futaki et al., 2013; Kolano et al., 2021*)*, where higher positive charges improve the uptake, probably due to their enhanced interaction with the negatively charged bacterial membrane composed of lipopolysaccharides. The ionic interactions allow better accumulation of the peptides on the bacterial surface and facilitate passive diffusion into the periplasm prior to transporter-mediated uptake (Lohner and Blondelle, 2005). However, the cell wall and capsule of *C. neoformans* differ significantly from those of bacteria. Nonetheless, a common feature is the negative charge of the outermost layer, the capsule, which is not composed of LPS in *C. neoformans*, but of complex polysaccharides, such as glucuronoxylomannan (GXM) and glucuronoxylomannogalactan (GXMGal) (O'Meara and Alspaugh, 2012; Casadevall et al., 2019). Thus, cationic AMPs with a higher net charge can interact more strongly with the capsule, resulting in increased peptide accumulation on the surface that may improve penetration into the cell. Another significant difference is the composition of the cell wall, which includes various glucans, chitin, chitosan, mannoproteins, and most importantly, the characteristic melanin layer (Garcia-Rubio et al., 2019). Melanin is a hydrophobic, negatively charged polymer of phenolic and/or indolic compounds, also known as the “antifungal resistance factor” because it reduces susceptibility to antifungal drugs (Ikeda et al., 2003; Nosanchuk and Casadevall, 2006). Incubation of melanin with amphotericin B significantly reduced its activity against *C. neoformans*, while the small molecules fluconazole and flucytosine remained unaffected (Casadevall et al., 2000). Our studies showed that Api88, Api795, Onc112, and Chex1Arg20 were able to enter the fungal cytoplasm after passing the fungal cell wall and capsule including the melanin layer. It can be speculated that both the positive charge of the peptides and a certain degree of hydrophobicity are advantageous, which may be caused by repeated Pro-Arg-Pro motifs, which are characteristic of insect-derived PrAMPs. The observed differences in activity among the PrAMPs tested are most likely due to differences in charge and basicity rather than hydrophobicity, as they differ only slightly in this respect. The natural PrAMPs apidaecin 1b, drosocin, and pyrrhocoricin were inactive against *C. neoformans*, possibly due to their lower net charge compared to the optimized derivatives, which likely results in poorer uptake. However, it should be noted that charge alone does not explain the differences in activity, as seen for Onc112 and Onc72 and especially for the scrambled peptides. There must be further structural features important for the antifungal activity in order to obtain an ideal interplay between uptake, target binding, and target inhibition.

## 5 Conclusion

Bovine and especially short insect-derived PrAMPs have been extensively studied and rationally optimized for potential systemic therapeutic antibacterial treatments over the last decades due to their high protease stability and low intrinsic toxicity, such as the development of the designer peptides Api88, Api137, Onc72, Onc112, and Chex1Arg20 with proven efficacy in various murine bacterial infection models. Here, we showed for the first time that these peptides are also highly active against several clinically relevant *C. neoformans* strains that are difficult to treat with current antifungal agents. The observed MICs were as low as previously reported for the most susceptible Gram-negative bacteria, i.e., *E. coli* and *K. pneumoniae*. Interestingly, reverse sequences were similarly active, while scrambled sequences were essentially inactive. Fluorescence microscopy revealed that the peptides enter the cytoplasm of apparently intact cells in larger quantities, most likely ruling out a membranolytic mechanism, and suggesting an intracellular target, possibly the fungal ribosome in analogy to the established mode of action in Gram-negative bacteria. However, the different composition and structure of the bacterial and fungal ribosomes requires confirmation of the proposed target, as the active peptides could by chance hit another target resulting in a different mechanism. However, scanning electron microscopy showed that PrAMPs also have a slight damaging effect on the cell exterior, which could facilitate entry into the cell interior.

## Data availability statement

The original contributions presented in the study are included in the article/Supplementary material, further inquiries can be directed to the corresponding author.

## Author contributions

AB: Conceptualization, Formal analysis, Investigation, Methodology, Visualization, Writing – original draft. TG: Formal analysis, Investigation, Methodology, Visualization, Writing – review & editing. SF: Investigation, Writing – review & editing, Methodology. DK: Investigation, Writing – review & editing. AK: Methodology, Writing – review & editing. SAF: Methodology, Resources, Writing – review & editing. GA: Resources, Writing – review & editing. RH: Conceptualization, Methodology, Resources, Writing – review & editing. UM: Conceptualization, Investigation, Methodology, Writing – review & editing.
