# Supplementary material for: Evaluation of proline-rich antimicrobial peptides as potential lead structures for novel antimycotics against Cryptococcus neoformans
Source: Front Microbiol. 2024 Jan 8;14:1328890. doi: 10.3389/fmicb.2023.1328890 (PMC10800876; doi:10.3389/fmicb.2023.1328890)
Supplement: Supplementary file 1 [file Data_Sheet_1.docx]

Supplementary Material

**Evaluation of proline-rich antimicrobial peptides as potential lead structures for novel antimycotics against *Cryptococcus neoformans***

Alexandra Brakel, Thomas Grochow, Stefanie Fritsche, Daniel Knappe, Andor Krizsan, Simone A. Fietz, Gottfried Alber, Ralf Hoffmann, and Uwe Müller

Table of contents

Table S1: MICs of tested peptides S1

Table S2: CFU counts after peptide treatment S2

Table S3: MICs of Cf-labeled peptides S3

Figure S1: Antifungal effect after peptide treatment S4

Figure S2: Antifungal effect of reversed peptides S5

Figure S3: Confocal Laser Scanning Microscope S6

Figure S4: Scanning Electron Microscopy S7

Figure S5: Hemolytic activity S8

Figure S6: Nucleotide release/leakage assay S9

Method M1: Nucleotide release/leakage assay S10

**Table S1:** Minimum inhibitory concentrations (MICs) of tested peptides are sequence dependent. MICs were determined for various peptides and antimycotics against the *C. neoformans* strains 1841, H99, and KN99α. The MIC values were determined twice at 24 h and 48 h. Shown is the mean of six replicates (*n* = 6) performed as triplicates on two separate days. MICs are expressed in mg/L. MICs expressed in µmol/L are listed additionally in Table 2.

|  | **MIC (mg/L)** | | | | | |
| --- | --- | --- | --- | --- | --- | --- |
|  | **1841** | | **H99** | | **KN99α** | |
| **Peptide** | **24 h** | **48 h** | **24 h** | **48 h** | **24 h** | **48 h** |
| **Api88** | 8 | 16 | 16 | 8 | 8 | 16 |
| **Api88 rev** | 16 | 16 | 16 | 32 | 16 | 32 |
| **Api88 rev*** | 8 | 8 | 8 | 8 | 8 | 8 |
| **Api88 scr** | 16 | 32-64 | 64 | 128 | 64 | 128 |
| **Api137** | 32 | 64 | 32 | 64 | 32-64 | 64-128 |
| **Api795** | 4 | 8 | 8 | 8 | 4-8 | 8 |
| **Api813** | 8 | 8 | 4 | 4-8 | 4-8 | 8 |
| **Api822** | 8 | 8 | 8 | 8 | 8 | 8 |
| **Apidaecin 1b** | > 128 | > 128 | > 128 | > 128 | > 128 | > 128 |
| **Apidaecin 1b scr** | > 128 | > 128 | > 128 | > 128 | > 128 | > 128 |
| **Onc72** | 64 | 128 | 32 | 64-128 | 32-64 | 128 |
| **Onc72 rev** | 64 | 128 | 64 | 128 | 64 | 128 |
| **Onc112** | 8 | 16 | 8-16 | 16 | 8-16 | 16-32 |
| **Chex1Arg20** | 4 | 8 | 4 | 4-8 | 4 | 8 |
| **Chex1Arg20 rev** | 8 | 8 | 8 | 8-16 | 8 | 16 |
| **Chex1Arg20 scr** | 32-64 | 64-128 | 64 | 128 | 64 | 128 |
| **Chex1Arg20 D4K** | 4 | 4 | 4 | 4 | 4 | 4 |
| **Drosocin** | 64 | 128 | 128 | >128 | >128 | >128 |
| **Pyrrhocoricin** | >128 | >128 | >128 | >128 | >128 | >128 |
| **Bac7 1-60** | 8 | 8 | 8 | 8 | 8 | 8-16 |
| **CRAMP** | 8 | 8 | 8 | 8 | 8 | 8 |
| **CRAMP rev** | 4 | 8 | 4 | 8 | 4-8 | 8 |
| **Amphotericin B** | 0.06-0.12 | 0.12 | < 0.02 | 0.04-0.08 | 0.08 | 0.16 |
| **Fluconazole** | 32 | 32 | 16 | 16-32 | 4 | 8 |
| **Flucytosine** | 64 | 128 | 64 | > 128 | 128 | > 128 |

**Table S2:** Cell counts of *C. neoformans* 1841, H99, and KN99α after treatment (3 h, 30 °C) with AMPs and antifungal agents corresponding to relative cell viability values of Figure 1. Aliquots of samples were spread out on SAB agar and CFU were counted after 24 h and 48 h. Shown is the mean of four replicates (n = 4) performed as duplicates on two separate days and the associated standard deviation.

| **peptide/compound** | **concentration (µmol/L)** | **cell count (x 10^4^ CFU/mL)** | | | | | |
| --- | --- | --- | --- | --- | --- | --- | --- |
|  |  | ***C. neoformans* 1841** | | ***C. neoformans* H99** | | ***C. neoformans* KN99α** | |
|  |  | **24 h** | **48 h** | **24 h** | **48 h** | **24 h** | **48 h** |
| Control | 0 | 2.6 ± 0.2 | 2.6 ± 0.2 | 5.0 ± 0.8 | 5.1 ± 0.7 | 3.5 ± 0.6 | 3.5 ± 0.7 |
| Amphotericin B | 0.25 | 1.4 ± 0.3 | 1.4 ± 0.3 | 0.9 ± 0.7 | 1.1 ± 0.7 | 2.4 ± 1.5 | 2.5 ± 1.7 |
|  | 2.5 | 0.0 ± 0.0 | 0.0 ± 0.0 | 0.0 ± 0.0 | 0.0 ± 0.0 | 0.1 ± 0.1 | 0.1 ± 0.1 |
|  | 25 | 0.0 ± 0.0 | 0.0 ± 0.0 | 0.0 ± 0.0 | 0.0 ± 0.0 | 0.0 ± 0.0 | 0.0 ± 0.0 |
| Fluconazole | 0.25 | 2.5 ± 0.0 | 2.5 ± 0.0 | 3.4 ± 0.5 | 3.8 ± 0.3 | 2.9 ± 0.5 | 3.0 ± 0.6 |
|  | 2.5 | 2.7 ± 0.2 | 2.7 ± 0.2 | 3.4 ± 0.4 | 3.5 ± 0.4 | 3.3 ± 0.6 | 3.4 ± 0.7 |
|  | 25 | 1.9 ± 0.3 | 1.9 ± 0.3 | 3.3 ± 0.8 | 3.5 ± 0.8 | 3.2 ± 0.5 | 3.3 ± 0.5 |
| Flucytosine | 0.25 | 2.2 ± 0.1 | 2.2 ± 0.1 | 3.1 ± 0.8 | 3.2 ± 0.7 | 3.4 ± 0.7 | 3.4 ± 0.8 |
|  | 2.5 | 2.4 ± 0.2 | 2.4 ± 0.2 | 3.7 ± 0.4 | 3.9 ± 0.3 | 3.1 ± 0.5 | 3.2 ± 0.5 |
|  | 25 | 2.4 ± 0.0 | 2.4 ± 0.0 | 3.1 ± 0.5 | 3.3 ± 0.6 | 3.3 ± 0.5 | 3.3 ± 0.5 |
| Apidaecin 1b | 0.25 | 1.9 ± 0.0 | 1.9 ± 0.0 | 3.8 ± 1.9 | 3.9 ± 1.9 | 3.0 ± 0.7 | 3.1 ± 0.8 |
|  | 2.5 | 1.5 ± 0.1 | 1.5 ± 0.1 | 3.9 ± 0.2 | 4.2 ± 0.2 | 2.9 ± 0.7 | 3.0 ± 0.8 |
|  | 25 | 1.2 ± 0.1 | 1.2 ± 0.1 | 3.2 ± 0.4 | 3.4 ± 0.3 | 2.7 ± 0.4 | 2.8 ± 0.5 |
| Api88 | 0.25 | 2.4 ± 0.1 | 2.4 ± 0.1 | 2.7 ± 0.4 | 2.8 ± 0.5 | 3.0 ± 0.7 | 3.1 ± 0.8 |
|  | 2.5 | 1.0 ± 0.1 | 1.0 ± 0.1 | 3.0 ± 0.2 | 3.3 ± 0.3 | 3.0 ± 0.6 | 3.1 ± 0.7 |
|  | 25 | 0.1 ± 0.0 | 0.1 ± 0.0 | 1.7 ± 0.2 | 2.0 ± 0.4 | 1.5 ± 0.2 | 1.6 ± 0.3 |
| Api795 | 0.25 | 1.4 ± 0.6 | 1.4 ± 0.6 | 3.3 ± 0.3 | 3.6 ± 0.6 | 2.9 ± 0.3 | 2.9 ± 0.4 |
|  | 2.5 | 2.1 ± 0.1 | 2.1 ± 0.1 | 1.4 ± 0.4 | 2.0 ± 0.2 | 1.7 ± 0.7 | 1.9 ± 0.8 |
|  | 25 | 0.2 ± 0.0 | 0.2 ± 0.0 | 0.1 ± 0.0 | 0.5 ± 0.2 | 0.1 ± 0.1 | 0.4 ± 0.1 |
| Onc112 | 0.25 | 2.4 ± 0.3 | 2.6 ± 0.3 | 3.8 ± 0.8 | 4.0 ± 0.8 | 2.7 ± 0.6 | 2.9 ± 0.7 |
|  | 2.5 | 1.8 ± 0.1 | 2.0 ± 0.2 | 2.8 ± 0.5 | 2.9 ± 0.4 | 2.7 ± 1.1 | 2.8 ± 1.2 |
|  | 25 | 0.1 ± 0.0 | 0.9 ± 0.2 | 0.1 ± 0.1 | 1.1 ± 0.1 | 0.1 ± 0.0 | 0.8 ± 0.2 |
| Chex1Arg20 | 0.25 | 2.7 ± 0.0 | 2.8 ± 0.1 | 1.4 ± 0.7 | 1.6 ± 0.8 | 2.7 ± 0.4 | 2.7 ± 0.4 |
|  | 2.5 | 0.0 ± 0.0 | 0.5 ± 0.1 | 1.5 ± 1.0 | 2.5 ± 0.8 | 1.0 ± 0.6 | 1.8 ± 1.2 |
|  | 25 | 0.0 ± 0.0 | 0.0 ± 0.0 | 0.0 ± 0.0 | 0.8 ± 0.2 | 0.5 ± 0.8 | 0.8 ± 0.9 |
| Chex1Arg20 D4K | 0.25 | 0.4 ± 0.0 | 2.2 ± 0.3 | 2.7 ± 0.4 | 2.9 ± 0.4 | 2.0 ± 1.2 | 2.2 ± 1.1 |
|  | 2.5 | 0.0 ± 0.0 | 0.0 ± 0.0 | 0.0 ± 0.0 | 0.1 ± 0.0 | 0.1 ± 0.1 | 0.1 ± 0.1 |
|  | 25 | 0.0 ± 0.0 | 0.0 ± 0.0 | 0.0 ± 0.0 | 0.0 ± 0.0 | 0.8 ± 1.3 | 0.8 ± 1.3 |
| CRAMP | 0.25 | 1.0 ± 0.0 | 1.1 ± 0.0 | 3.4 ± 0.6 | 3.5 ± 0.6 | 2.5 ± 1.4 | 2.5 ± 1.4 |
|  | 2.5 | 0.0 ± 0.0 | 0.0 ± 0.0 | 0.1 ± 0.1 | 0.2 ± 0.1 | 0.7 ± 0.5 | 0.7 ± 0.4 |
|  | 25 | 0.0 ± 0.0 | 0.0 ± 0.0 | 0.0 ± 0.0 | 0.0 ± 0.0 | 0.0 ± 0.0 | 0.0 ± 0.0 |

**Table S3:** Minimum inhibitory concentrations (MICs) determined for peptides labeled with 5(6)-carboxyfluorescein (Cf) against *C. neoformans* 1841. The MIC values were determined twice at 24 h and 48 h. Shown is the mean of three replicates (*n* = 3).

| **Peptide** | **MIC (mg/L)** | |
| --- | --- | --- |
|  | **24 h** | **48 h** |
| Cf-Api88 | 32 | 32-64 |
| Cf-Api137 | > 64 | > 64 |
| Cf-Api795 | 4 | 4 |
| Cf-Apidaecin 1b | >128 | >128 |
| Cf-Onc112 | 4-8 | 4-8 |
| Cf-Chex1Arg20 | 4 | 4 |
| Cf-Chex1Arg20 D4K | 8 | 8 |


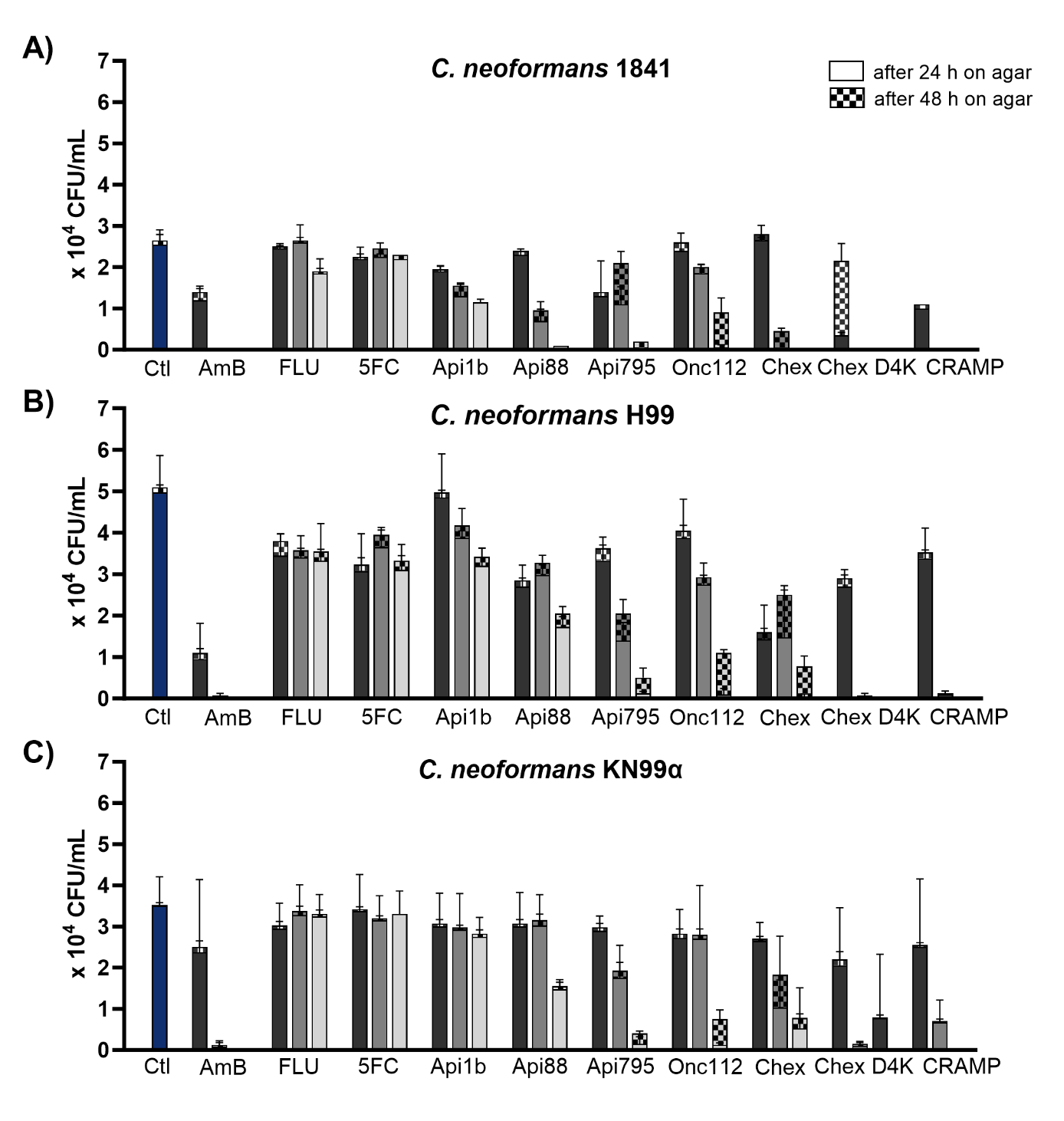


**Figure S1:** PrAMPs and the cathelicidin CRAMP have a concentration-dependent effect on cell viability and are able to reduce colony-forming units. CFU of *C. neoformans* strains A) 1841, B) H99, and C) KN99α was assessed after incubation (3 h, 30 °C) with AMPs and antifungals at concentrations of 0.25 µmol/L (darker gray), 2.5 µmol/L (gray), and 25 µmol/L (lighter gray). Divided bars show colony-forming units (CFU) counted after 24 h (no pattern) and 48 h (checkered) incubation at 30 °C. Shown is the mean of four replicates (*n* = 4) performed as duplicates on two separate days and the associated standard deviation. Ctl – control; AmB – Amphotericin B; FLU – fluconazole; 5FC – flucytosine; Chex – Chex1Arg20; Chex D4K – Chex1Arg20 D4K.


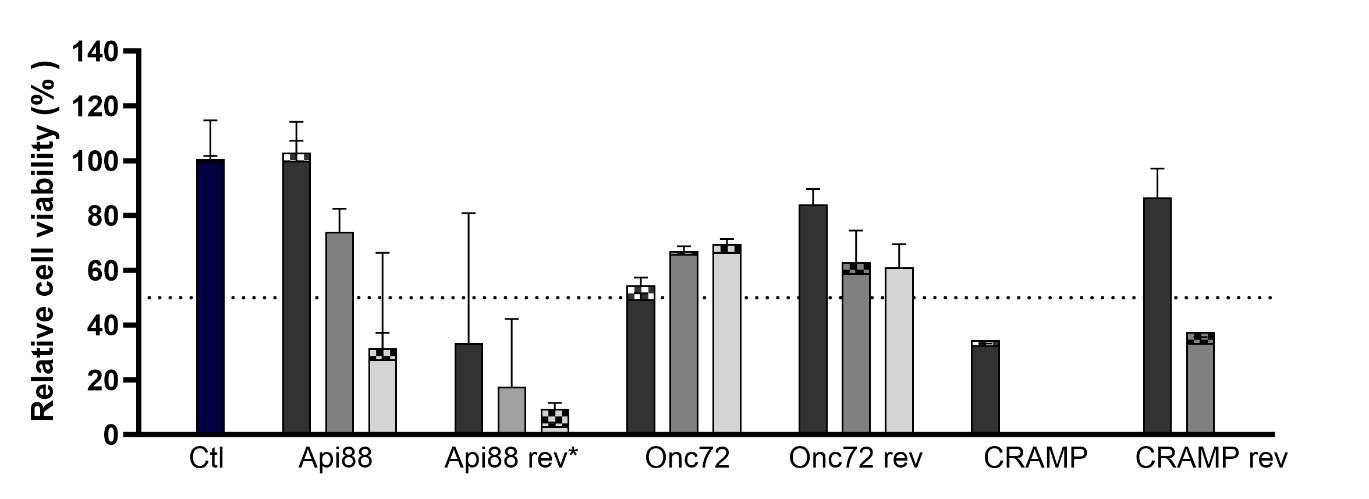


**Figure S2:** Effect of original and reverse peptide sequences of Api88 (* indicates that reversed version is elongated with PR-motif, listed in Table 1), Onc72, and CRAMP on antifungal activity against *C. neoformans* 1841. Fungal cells were incubated with 25 µmol/L (darker greay), 2.5 µmol/L (gray), and 0.25 µmol/L (lighter gray) peptide for 3 hours. Diluted cell suspensions were plated out on SAB agar and CFU were determined after 24 hours (no pattern) and 48 hours (checkered), shown as divided bars. Samples were normalized to the control sample (Ctl, no peptide or antifungal added, blue). Dotted line indicates 50 % inhibition. Shown is the mean of four replicates (*n* = 4) performed as duplicates on two separate days and the associated standard deviation.


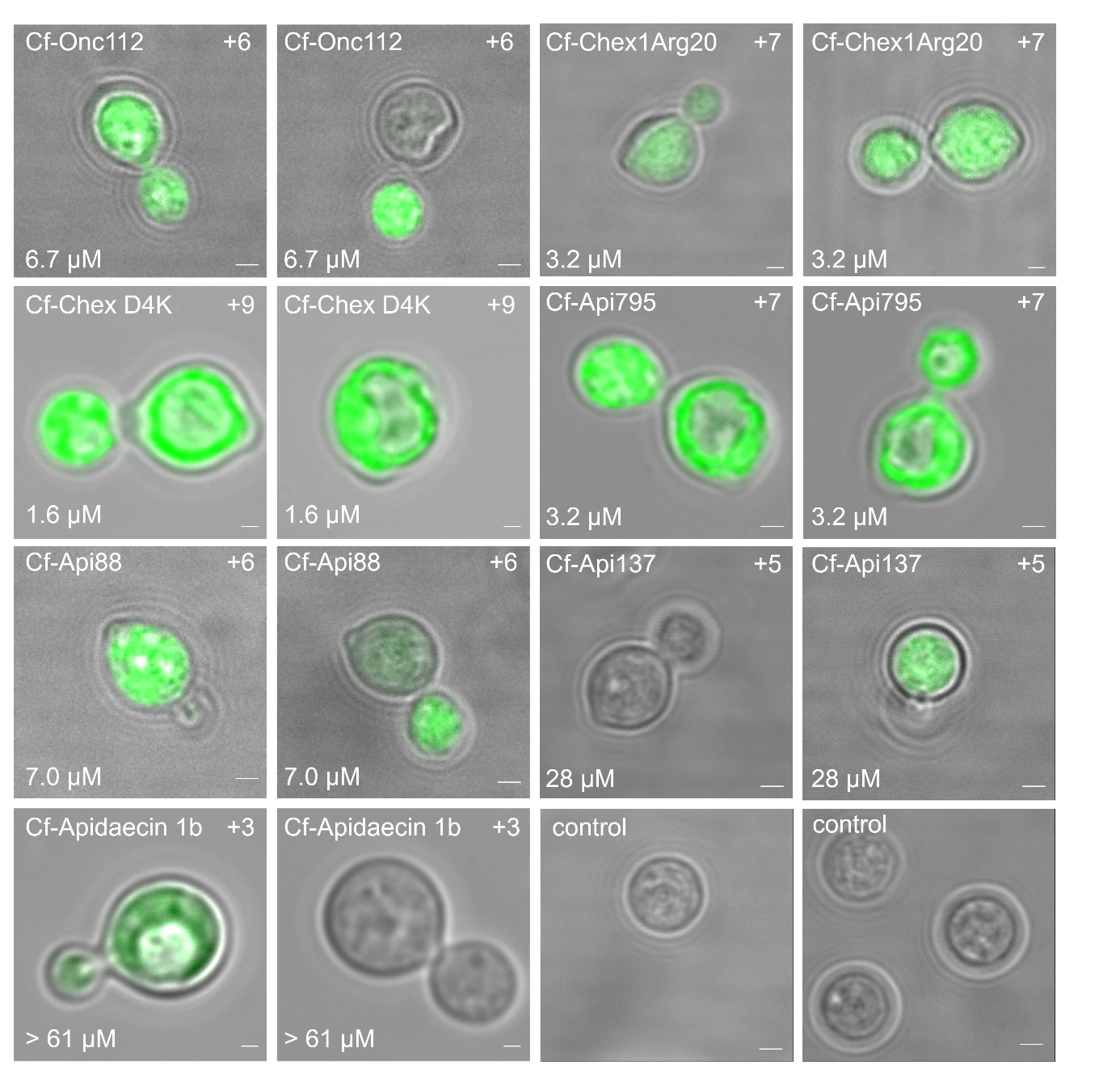


**Figure S3:** Confocal laser scanning images of *C. neoformans* 1841 cells incubated with Cf-labeled peptides (25 µmol/L) at 30 °C for 3 h. Cells were immobilized on glass slides and fluorescence was measured for the entire cell (λ_ex_ = 496 nm, λ_em_ = 503-600 nm). For each peptide, the central z-stack was shown as an overlay of the white light image and fluorescence channel. The MICs (after 48 h, lower left corner) and net charge (upper right corner) are provided for each peptide as well. Scale bar, 1 µm.


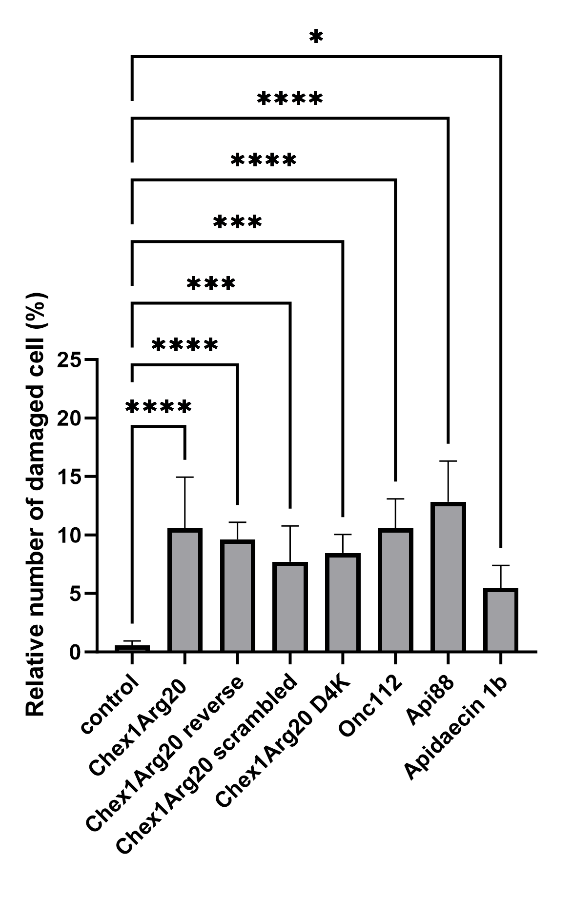


**Figure S4:** Scanning electron microscopy images show a percentage of cells with damaged membrane after peptide incubation. Cells (10^8^ CFU/mL) were incubated with peptide concentrations of 200 µmol/L for 3 h. An untreated sample served as a control. All samples shown were treated under the same conditions. Damaged and intact cells from five SEM images were counted per peptide, and the relative proportion of damaged cells was calculated. Data represent the mean including the standard deviation (*n* = 5). Data were analyzed using Dunnett's multiple comparisons test (GraphPad Prism 10.0.3, **** *P* < 0.0001, *** *P* < 0.001, * *P* < 0.1).


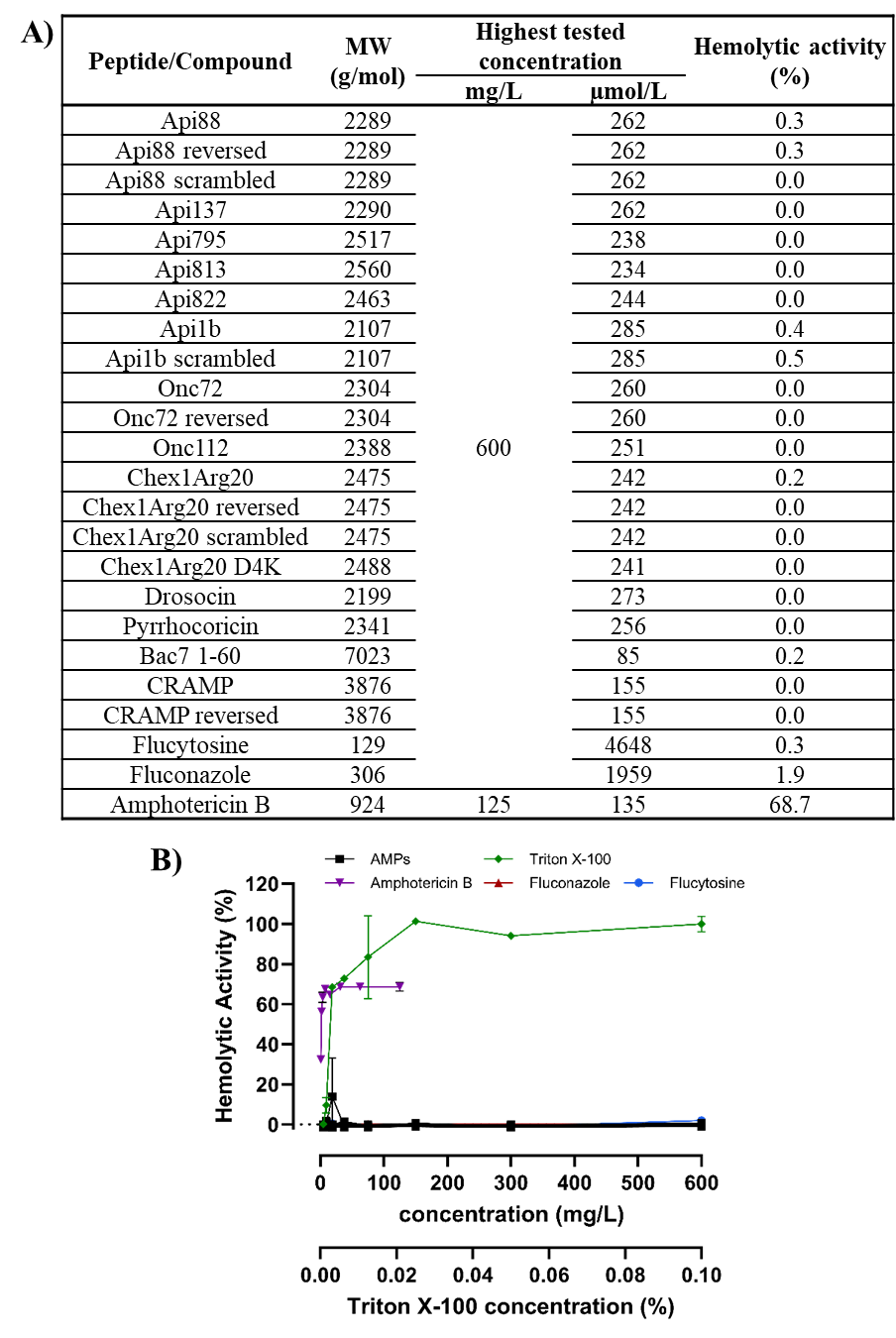


**Figure S5:** PrAMPs did not show a hemolytic activity, even for the highest concentration (600 mg/L) tested (A). Porcine erythrocytes were incubated with a serial dilution of peptides or antifungal agents from 600 to 5 mg/L (Amphotericin B from 125 mg/L to 1 mg/L) at 37 °C for 1 h (B). The optical density at 405 nm of the supernatant was measured. Hemolytic activity was normalized to the optical density at 405 nm of 0.1% Triton X-100 (set to 100 %) serving as a positive control.


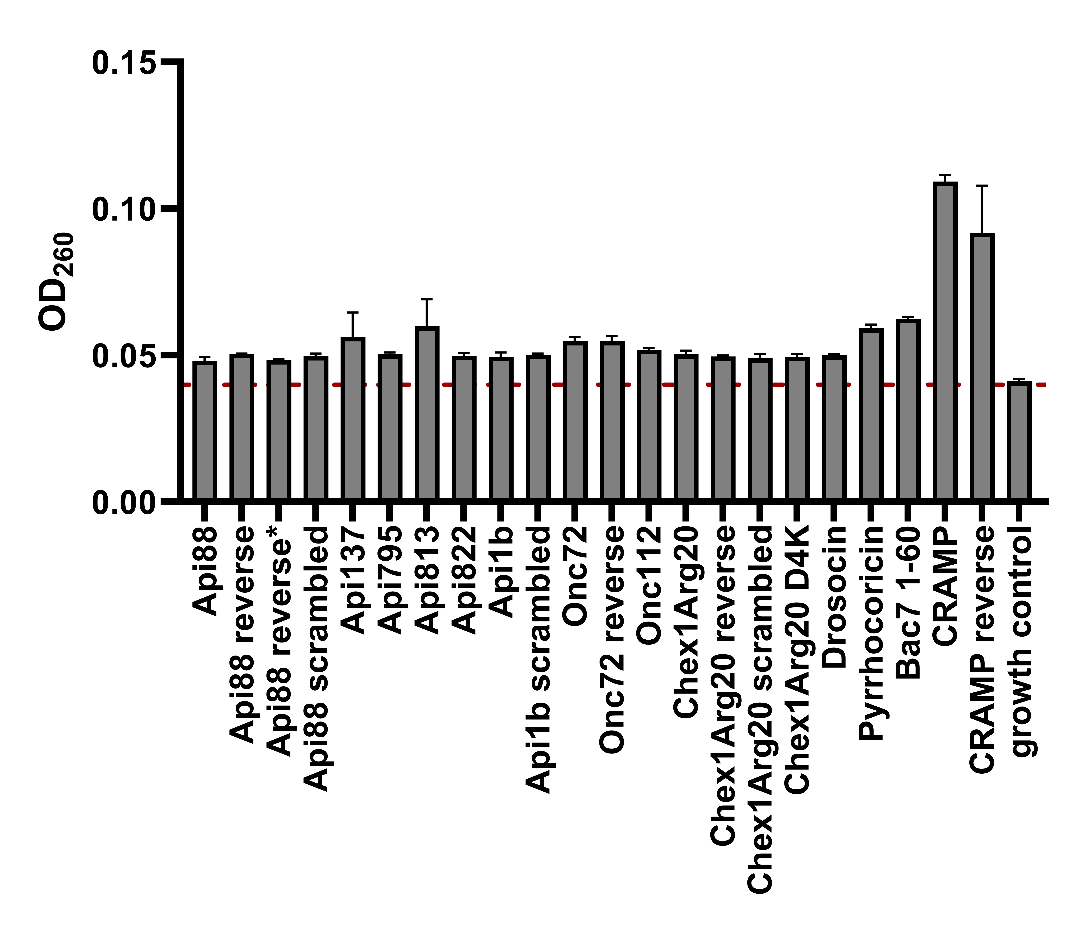


**Figure S6:** PrAMP treatment did not induce nucleotide release from *C. neoformans* 1841. The nucleotide release was determined by measuring of the optical density at 260 nm (Supplement, Method M1). An untreated sample incubated with phosphate buffer served as growth control. All samples were incubated for 3 h at 30 °C. The mean of triplicates and the corresponding standard deviation are shown.

**Method M1:** Nucleotide Leakage Assay

Nucleotide release was measured using recently reported protocols with minor modifications (1, 2). *C. neoformans* 1841 was grown in Sabouraud medium (SAB, 2 % glucose, 1 % peptone) overnight at 30 °C on an orbital shaker (80 rpm). The sample was centrifuged (10 min, 4 °C, 400 × g, Allegra X-22R, Rotor SX4250, Beckmann Coulter, Krefeld, Germany) and the cell pellet was washed with phosphate buffer (10 mmol/L Na_2_HPO_4_/NaH_2_PO_4_, pH 7.5). After a second centrifugation step, the cells were resuspended and adjusted to 2 × 10^6^ CFU/mL with phosphate buffer. Aqueous peptide solutions (3 g/L) were diluted to 50 µmol/L with phosphate buffer to achieve a final concentration of 25 µmol/L in the well (50 µL/well; polystyrene V-bottom, Greiner Bio-One GmbH). An untreated sample of *C. neoformans* 1841 in phosphate buffer was used as a reference. Cell culture suspension (50 µL/well) was added and the plate was incubated for 3 h at 30 °C on an orbital shaker. The plate was centrifuged (10 min, 1000 × g, Allegra X-22R, Rotor SX4250, Beckmann Coulter) and the supernatant was transferred to a second 96-well plate (UV Star F-bottom, Greiner Bio-One GmbH, Frickenhausen, Germany). Optical density at 260 nm was measured using a microplate reader (PARADIGM^TM^, Molecular Devices). The experiment was performed in triplicate.

1. Lemos AS, Florêncio JR, Pinto NC, Campos LM, Silva TP, Grazul RM, et al. Antifungal Activity of the Natural Coumarin Scopoletin Against Planktonic Cells and Biofilms From a Multidrug-Resistant Candida tropicalis Strain. *Front Microbiol* (2020) **11**:1525. doi:10.3389/fmicb.2020.01525

2. Yang L, Tian Z, Zhou L, Zhu L, Sun C, Huang M, et al. In vitro Antifungal Activity of a Novel Antimicrobial Peptide AMP-17 Against Planktonic Cells and Biofilms of Cryptococcus neoformans. *Infect Drug Resist* (2022) **15**:233–48. doi:10.2147/IDR.S344246
